# Tau activation of microglial cGAS–IFN reduces MEF2C-mediated cognitive resilience

**DOI:** 10.1038/s41593-023-01315-6

**Published:** 2023-04-24

**Authors:** Joe C. Udeochu, Sadaf Amin, Yige Huang, Li Fan, Eileen Ruth S. Torres, Gillian K. Carling, Bangyan Liu, Hugo McGurran, Guillermo Coronas-Samano, Grant Kauwe, Gergey Alzaem Mousa, Man Ying Wong, Pearly Ye, Ravi Kumar Nagiri, Iris Lo, Julia Holtzman, Carlo Corona, Allan Yarahmady, Michael T. Gill, Ravikiran M. Raju, Sue-Ann Mok, Shiaoching Gong, Wenjie Luo, Mingrui Zhao, Tara E. Tracy, Rajiv R. Ratan, Li-Huei Tsai, Subhash C. Sinha, Li Gan

**Affiliations:** 1 Helen and Robert Appel Alzheimer’s Disease Research Institute, Feil Family Brain and Mind Research Institute, Weill Cornell Medicine, New York, NY USA; 2 The Gladstone Institute of Neurological Disease, San Francisco, CA USA; 3 Buck Institute for Research on Aging, Novato, CA USA; 4 Burke Neurological Institute at Weill Cornell Medicine, White Plains, NY USA; 5 Department of Biochemistry, University of Alberta, Edmonton, Alberta Canada; 6 The Picower Institute of Learning and Memory, Department of Brain and Cognitive Sciences, Massachusetts Institute of Technology, Cambridge, MA USA; 7 Division of Newborn Medicine, Boston Children’s Hospital, Boston, MA USA

**Keywords:** Alzheimer's disease, Neuroimmunology

## Abstract

Pathological hallmarks of Alzheimer’s disease (AD) precede clinical symptoms by years, indicating a period of cognitive resilience before the onset of dementia. Here, we report that activation of cyclic GMP–AMP synthase (cGAS) diminishes cognitive resilience by decreasing the neuronal transcriptional network of myocyte enhancer factor 2c (MEF2C) through type I interferon (IFN-I) signaling. Pathogenic tau activates cGAS and IFN-I responses in microglia, in part mediated by cytosolic leakage of mitochondrial DNA. Genetic ablation of *Cgas* in mice with tauopathy diminished the microglial IFN-I response, preserved synapse integrity and plasticity and protected against cognitive impairment without affecting the pathogenic tau load. cGAS ablation increased, while activation of IFN-I decreased, the neuronal MEF2C expression network linked to cognitive resilience in AD. Pharmacological inhibition of cGAS in mice with tauopathy enhanced the neuronal MEF2C transcriptional network and restored synaptic integrity, plasticity and memory, supporting the therapeutic potential of targeting the cGAS–IFN–MEF2C axis to improve resilience against AD-related pathological insults.

## Abstract (web summary)

Mechanisms of cognitive resilience against Tau pathology are unknown. The authors show that inactivation of the microglial cGAS–interferon axis confers such resilience by preserving the neuronal expression of myocyte enhancer factor 2c.

## Main

Alzheimer’s disease (AD) is the most common late-onset dementia. A long preclinical asymptomatic period with increasing deposition of amyloid-β plaques and tau aggregates transforms to a symptomatic phase with cognitive decline^1–3^. While the transition is poorly understood, it coincides with alterations in innate immune responses, vasculature and metabolism^3^. Susceptibility to sporadic late-onset AD is linked to single-nucleotide polymorphisms in innate immune genes^4,5^, suggesting that maladaptive innate immune responses underlie the cognitive decline.

Antiviral response pathways are upregulated in AD and regulate microglial disease responses, including immune activation/suppression and synaptic pruning in aging and neurodegenerative diseases^6–9^. Cyclic GMP–AMP synthase (cGAS), the major antiviral DNA sensor, binds double-stranded DNA (dsDNA) and catalyzes cyclic GMP–AMP (cGAMP) formation, which activates stimulator of interferon (IFN) genes (STING). STING recruits tank binding kinase 1 (TBK1) and promotes TBK1 autophosphorylation to activate type I IFN (IFN-I) expression^10^. The cGAS–STING pathway drives deleterious IFN-I activation in neurodegenerative diseases, including Parkinson’s disease, amyotrophic lateral sclerosis and Huntington’s disease^11–13^. cGAS–STING activation exacerbates amyloid-β pathology in 5xFAD mice^14^. While tau activates the cGAS–STING pathway and increases NF-κB signaling in cultured microglia^15^, how cGAS activation renders the brain vulnerable to tau-related cognitive decline remains unknown.

Here, we investigated cGAS–STING–IFN activation in microglia in mice with tauopathy and in brain samples from individuals with AD. Using behavior, electrophysiology and single-nuclei RNA sequencing (snRNA-seq), we demonstrated potent protective effects of *Cgas* genetic deletion against synaptic and cognitive deficits associated with induction of the transcriptional network of myocyte enhancer factor 2c (*Mef2c*), a gene implicated in cognitive resilience^16^. snRNA-seq analyses of AD brains revealed dysregulation of microglial IFN-I genes and the neuronal MEF2C transcriptional network. We propose a mechanism of maladaptive innate immune responses that confers cognitive vulnerability and present therapeutic strategies to enhance resilience against AD-related dementia.

## Results

### cGAS–STING is activated in tau transgenic mice and human AD

**Fig. 1:**
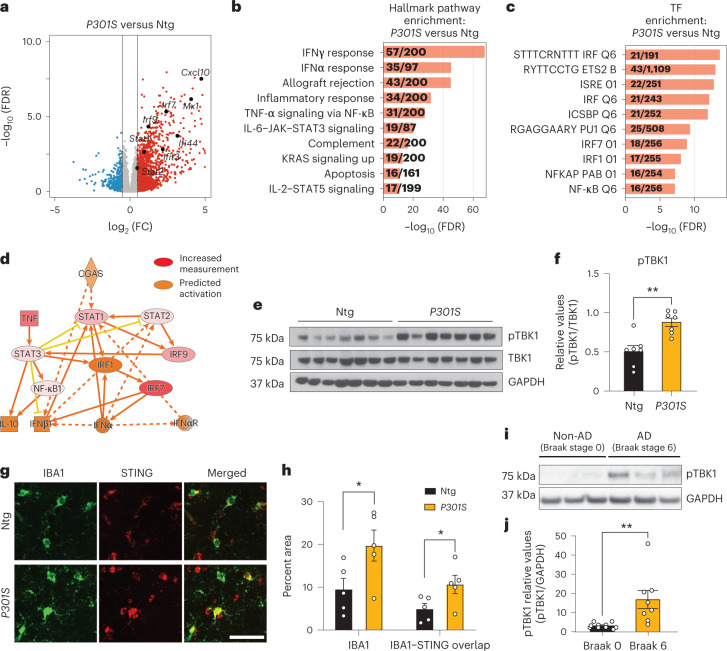
The cGAS–STING pathway is activated in the hippocampi of mice with tauopathy and in human AD brains. **a**, Volcano plot of RNA-seq data from bulk hippocampal tissue from 8- to 9-month-old *P301S* transgenic and non-transgenic mice (Wald test). Red and blue dots represent genes with a log_2_ FC (fold change) of > 0.5 and < −0.5, respectively. All other genes are colored gray. Selected upregulated IFN genes are labeled; *n* = 7 non-transgenic mice and *n* = 6 *P301S* transgenic mice; FDR, false discovery rate; Ntg, non-transgenic; FC, fold change. **b**, Gene set enrichment analysis showing hallmark pathways associated with the top 500 DEGs upregulated in *P301S* transgenic samples compared to in non-transgenic samples. **c**, Gene set enrichment analysis showing the top TFs associated with the top 500 DEGs upregulated in *P301S* transgenic samples compared to in non-transgenic samples. **d**, IPA prediction of cGAS as an upstream regulator of upregulated DEGs identified using an activation *z* score of >1 and a *P* value overlap of <0.05. **e**, Western blots for pTBK1, total TBK1 and GAPDH using hippocampal tissue lysates. Lanes 1–7: Ntg. Lanes 8–14: *P301S* transgenic. **f**, Ratio of pTBK1 to TBK1 from **e** showing significantly higher pTBK1 in *P301S* transgenic hippocampi than in non-transgenic hippocampi. Data are reported as mean ± s.e.m.; *n* = 7 animals per genotype; **P = 0.0015 two-tailed unpaired *t*-test. **g**, Representative immunofluorescence images of non-transgenic and *P301S* trasgenic hippocampi labeled with anti-IBA1 (green) and anti-STING (red); scale bar, 50 µm. **h**, Quantification of IBA1 and STING immunofluorescence intensities, showing increased IBA1 coverage and IBA1–STING overlap in *P301S* transgenic hippocampi. Results are presented as average intensity measurements from three to four sections per animal. Data are reported as mean ± s.e.m.; Ntg, *n* = 5; *P301S*, *n* = 5. IBA1: *P = 0.0498; IBA1–STING overlap: *P = 0.0497. Data were analyzed by two-tailed unpaired *t*-test. **i**, Representative western blots for pTBK1 and GAPDH using human frontal cortex brain lysates. Lanes 1–3: non-AD (Braak stage 0). Lanes 4–6: AD (Braak stage 6). **j**, Ratio of pTBK1 to GAPDH from **i** showing significantly higher pTBK1 in AD brains than in non-AD brains. Data are reported as mean ± s.e.m.; *n* = 10 non-AD brains and *n* = 8 AD brains; **P = 0.0054. Data were analyzed by two-tailed unpaired *t*-test. Source data

**Extended Data Fig. 1:**
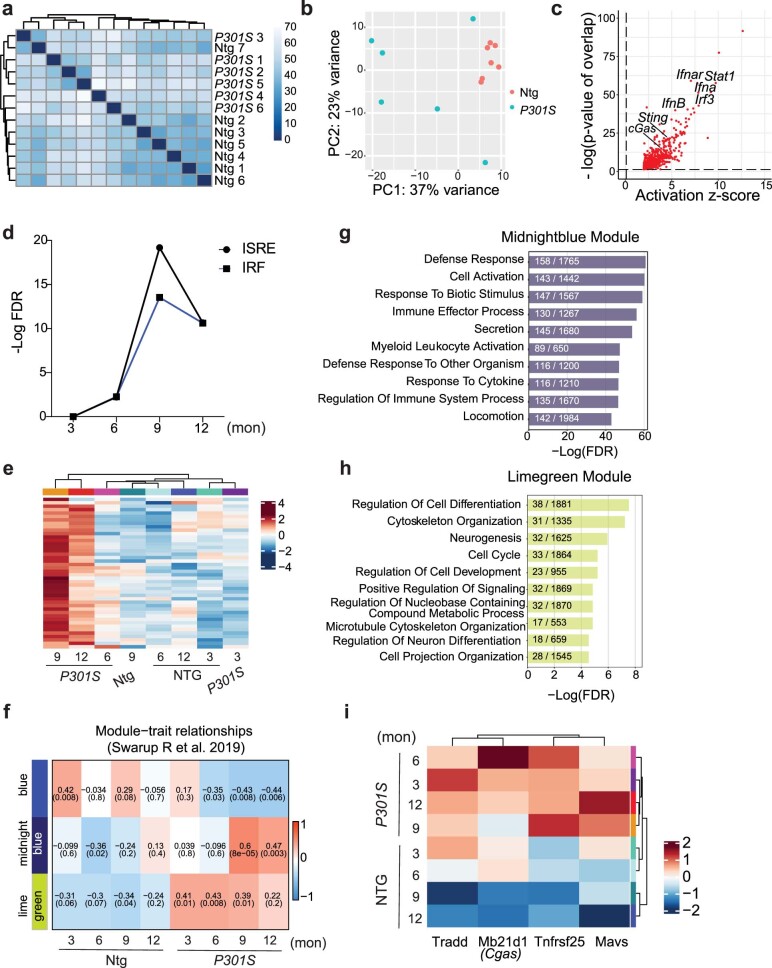
Temporal analysis of interferon gene signature in *P301S* mice (Related to Fig. 1). **a**. Hierarchical clustering of Non-transgenic (Ntg) and *P301S* hippocampal RNA counts. **b**. PCA plot showing distribution on Ntg and *P301S* samples according to variance driven by principal component 1 (PC1) and principal component 2 (PC2). **c**. Predicted upstream regulators associated with upregulated DEGs in *P301S* samples, from Ingenuity Pathway Analysis. *P*-value overlap < 0.01. Right-tailed Fisher’s Exact Test. **d**. Enrichment of interferon-sensitive response elements (ISREs) and interferon regulator factors (IRFs) among DEGs in 3-, 6-, 9-, and 12-month-old *P301S* hippocampi. **e**. Heatmap showing expression patterns of interferon DEGs from (**d**). **f**. Module-trait relationship heatmap derived from weighted gene correlation analyses showing correlation of gene expression modules with genotype and age. **g**. Top 10 Gene Ontology pathways overrepresented in midnight-blue module. **h**. Top 10 Gene Ontology pathways overrepresented in lime-green module. **i**. Cytosolic nucleotide-sensing genes, *Cgas* and *Mavs*, are components of the lime-green module.

To characterize gene expression changes in tauopathy, we performed bulk RNA-seq of *P301S* tau transgenic and non-transgenic hippocampi (8–9 months; Extended Data Fig. 1a,b). IFN genes were upregulated in *P301S* transgenic hippocampi compared to in non-transgenic hippocampi, and transcription factor (TF) motifs of IFN response factors (IRFs) and IFN-sensitive response elements (ISREs) were enriched (Fig. 1a–c and Supplementary Table 1). Ingenuity Pathway Analysis (IPA) of predicted regulators of upregulated differentially expressed genes (DEGs) confirmed components of IFN signaling (*Ifnar1*, *Stat1* and *Irf3*). Components of the cGAS–STING pathway (cGAS, STING and TBK1) were predicted activators of upregulated DEGs (Fig. 1d and Extended Data Fig. 1c). Immunoblotting for TBK1 phosphorylation revealed increased TBK1 phosphorylation in *P301S* transgenic hippocampal lysates (Fig. 1e,f). By immunofluorescent labeling, we further confirmed that microglial STING expression was increased in *P301S* transgenic hippocampi compared to in non-transgenic hippocampi (Fig. 1g,h).

Activation of cGAS–STING was further analyzed in previously published microarray profiles of hippocampi from 3-, 6-, 9- and 12-month-old *P301S* transgenic mice^17^. IRF and ISRE TF motifs were enriched in DEGs associated with tauopathy (Extended Data Fig. 1d,e). Using weighted gene correlation analysis^18^, we assessed the temporal nature of expression changes in *P301S* transgenic mice and identified three distinct gene expression modules: blue, midnight blue and lime green (Extended Data Fig. 1f)^18^. Lime green and midnight blue modules were positively associated with transgene expression (Supplementary Table 2). Notably, lime green (up at all ages) was enriched with terms such as cytoskeleton organization and cell cycle, reflecting early disease (Extended Data Fig. 1g,h)^19^. *Cgas* and mitochondrial antiviral signaling protein (*Mavs*) genes were part of the lime green module, implicating cytosolic nucleic acid-sensing pathways in the induction of innate immune responses of tauopathy (Extended Data Fig. 1i).

IFN signaling is upregulated in AD brains^6–8^. To determine cGAS involvement in that response, we assessed phosphorylated TBK1 (pTBK1) levels in healthy and AD (Braak stage 0 versus 6) postmortem brain samples (Supplementary Table 3). pTBK1 levels were elevated in AD brains (Fig. 1i,j). Thus, the cGAS–STING pathway is activated in *P301S* transgenic mice with tauopathy and in AD brain samples.

### Tau induces mitochondrial DNA leakage and IFN activation

**Fig. 2:**
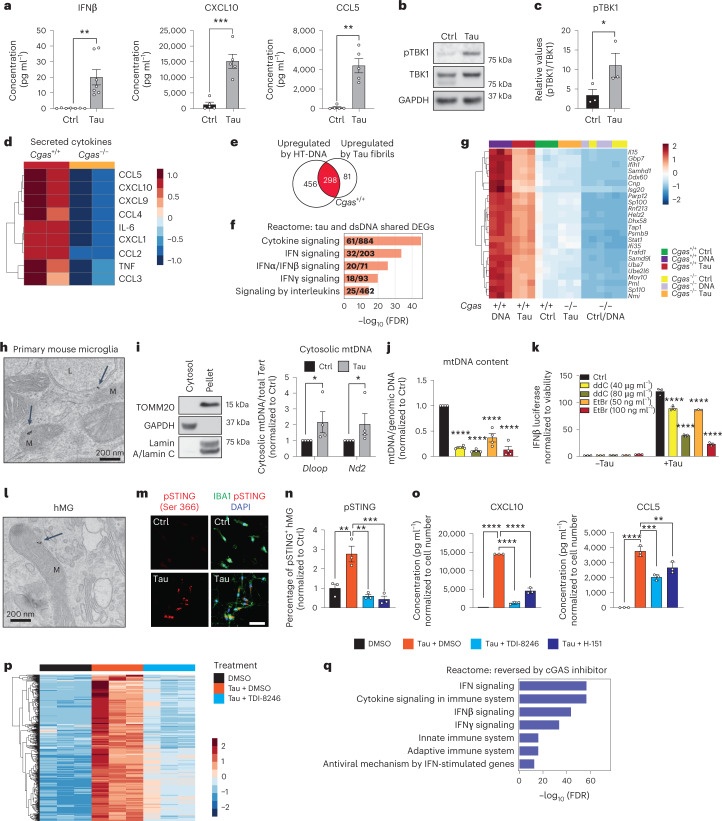
Tau stimulation induces a cGAS-dependent IFN response that partially depends on mitochondrial DNA leakage. **a**, Quantification of IFNβ expression by ELISA and CXCL10 and CCL5 expression by MAGPIX multiplex assay in culture medium supernatants from untreated (Ctrl) and tau-treated (Tau) primary mouse microglia. Data are reported as mean ± s.e.m. IFNβ: *n* = 7 independent primary microglial culture preparations treated with tau, **P = 0.0016; CXCL10 and CCL5: *n* = 5 independent primary microglial culture preparations treated with tau, ***P = 0.0004 and **P = 0.0031. Data were analyzed by two-tailed unpaired *t*-test. **b**, Representative western blots for pTBK1, total TBK1 and GAPDH using mouse primary microglial cell lysates (lane 1: untreated; lane 2: treated with tau fibrils). **c**, Ratio of pTBK1 to TBK1 from **b** showing significantly higher pTBK1 in tau fibril-treated primary microglia than in untreated microglia. Data are reported as mean ± s.e.m.; *n* = 3 independent primary microglial culture preparations treated with tau, *P = 0.0027. Data were analyzed by two-tailed unpaired *t*-test. **d**, Heat map showing the normalized levels of tau-induced cytokines in *Cgas*^+/+^ and *Cgas*^−/−^ primary cultured microglia. **e**, Bulk RNA-seq analysis for *Cgas*^+/+^ and *Cgas*^−/−^ primary cultured microglia treated or not treated with tau fibrils or HT-DNA; *n* = 3 per condition. Venn diagram showing the overlap of genes upregulated by HT-DNA and tau treatment in *Cgas*^+/+^ microglia; log_2_ FC > 1 and FDR < 0.05. **f**, Top five Reactome pathways represented in upregulated DEGs common to HT-DNA (dsDNA) and tau-treated *Cgas*^+/+^ microglia; FDR < 0.05. **g**, Heat map summary of IFN-stimulated genes that are lower in *Cgas*^−/−^ than in *Cgas*^+/+^ microglia stimulated with HT-DNA or tau. **h**, Electron micrograph of primary mouse microglia treated with tau fibrils and immunogold labeled with an antibody to tau; L, lysosome; M, mitochondria. The experiment was performed once, and tau particles were detected in multiple mitochondria from different fields (arrows). **i**, Left, western blot showing the absence of mitochondrial and nuclear markers in the cytosolic fraction. Right, quantification of cytosolic mtDNA by quantitative PCR (qPCR; *Dloop1*/*Tert* and *Nd2*/*Tert*) in cytosolic extracts of BV2 IFNβ–luciferase reporter cells treated with tau fibrils or untreated. Data are reported as mean ± s.e.m.; *n* = 4 biologically independent experiments; *P = 0.0286. Data were analyzed by two-tailed Mann–Whitney test. **j**, Ratio of mtDNA (*Dloop1*) to genomic DNA (*Tert*) measured by RT–qPCR on DNA extracts of BV2 IFNβ–luciferase reporter cells treated for 7 d with ddC (40 or 80 μg ml^–1^) or EtBr (50 or 100 ng ml^–1^) to generate mtDNA-depleted cells. The values are normalized to the untreated sample. Data are reported as mean ± s.e.m.; *n* = 4; ****P < 0.0001. Data were analyzed by one-way analysis of variance (ANOVA) followed by Dunnett’s multiple comparison test. Groups are color coded as in **k**. **k**, Control and mtDNA-depleted IFNβ–luciferase reporter BV2 cells were stimulated with and without tau fibrils. IFNβ signal and viability were measured 16 h later. IFNβ–luciferase signal is normalized to CellTiter-Glo signal to correct for viability/cell count. Data are reported as mean ± s.e.m.; *n* = 3 biologically independent samples; ****P < 0.0001. Data were analyzed by two-way ANOVA followed by a Sidak multiple comparison test. **l**, Electron micrograph of human iPSC-derived microglia treated with tau fibrils and immunogold labeled with an antibody to tau; M, mitochondria. The experiment was performed once, and tau particles were detected in multiple mitochondria from different fields (arrow). **m**, Immunostaining for phosphorylated STING (pSTING; Ser 366) and IBA1 in human iPSC-derived microglia treated with tau fibrils for 6 h or untreated. **n**, Quantification of the percentage of pSTING^+^ cells in human iPSC-derived microglia treated with tau fibrils and DMSO, 20 µM TDI-8246 and 2 µM H-151 for 18 h; *n* = 3. Data are reported as mean ± s.e.m.; *n* = 3 biologically independent samples. DMSO versus Tau + DMSO, **P = 0.0044; Tau + DMSO versus Tau + TDI-8256, **P = 0.0012; Tau + DMSO versus Tau + H-151, ***P = 0.0007. Data were analyzed by one-way ANOVA followed by a Tukey multiple comparison test. **o**, Quantification of CXCL10 and CCL5 protein expression by MAGPIX multiplex assay in culture medium supernatants from human iPSC-derived microglia treated with tau fibrils and DMSO, 20 µM TDI-8246 and 5 µM H-151; *n* = 3; **P < 0.01, ***P < 0.001, ****P < 0.0001. Data were analyzed by one-way ANOVA followed by a Tukey multiple comparison test. **p**, Heat map showing the expression of tau-inducible genes reversed by treatment with 20 µM TDI-8246 (a cGAS inhibitor); log_2_ FC > 0.5 or < 0.5 and FDR < 0.05. **q**, Gene set enrichment analysis showing Reactome pathways associated with tau-induced genes that were reversed by treatment with 20 µM TDI-8246; log_2_ FC > 0.5 and FDR < 0.05. Source data

**Extended Data Fig. 2:**
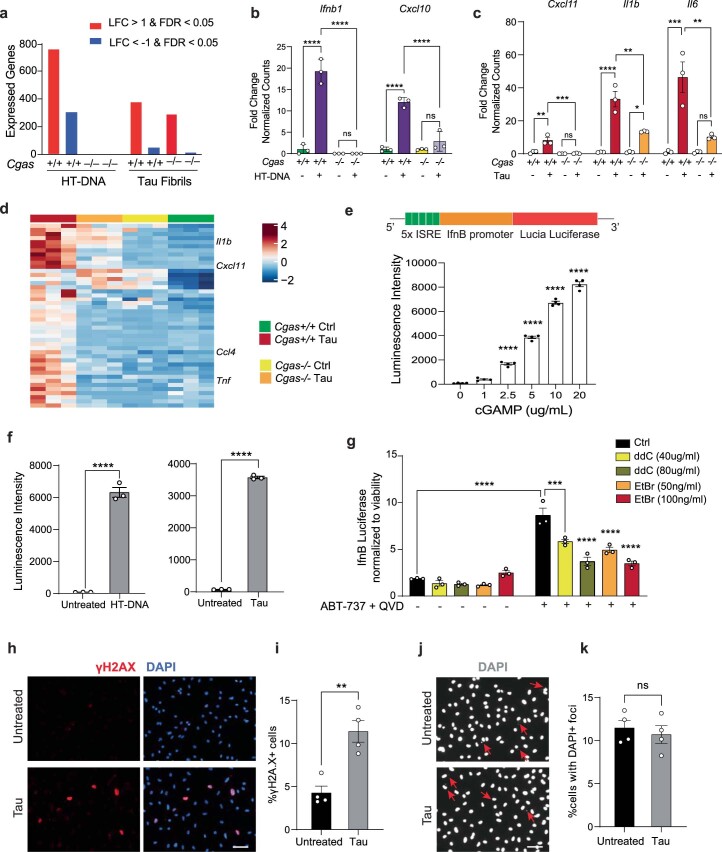
cGAS promotes IFN and proinflammatory cytokine signaling in tau-stimulated microglia (Related to Fig. 2). **a**. Summary of DEGs identified using DESeq2 (|log fold-change| > 1 and FDR < 0.05), grouped by genotype and treatment. **b**. Bar plots showing normalized counts of diminished *Ifnb1* and *Cxcl10* induction in HT-DNA-treated *Cgas*^*−/−*^ microglia. Data are reported as mean ± SD. n = 3 biologically independent samples. **** p < 0.0001. One-way ANOVA for each gene. **c**. Representative proinflammatory cytokine genes, *Cxcl11*, *Il1b* and *Il6*, that are significantly reduced in tau-stimulated *Cgas*^*-/-*^, compared to *Cgas*^*+/+*^ microglia. Data are reported as mean ± SEM. n = 3 biologically independent samples. *Cxcl11*: *Cgas* +/*+* vs *P301S Cgas* +/*+* ** p = 0.0018, *P301S Cgas* +/*+* vs *P301S Cgas−/−* *** p = 0.0009,,. *Il1b*: *Cgas* +/*+* vs *P301S Cgas* +*/+* **** p < 0.0001, *P301S Cgas* +/*+* vs *P301S Cgas−/−* ** p = 0.0016, *Cgas−/−* vs *P301S Cgas−/−* * p = 0.0241. *Il6*: *Cgas* +*/+* vs *P301S Cgas* +*/+* *** p = 0.0006, *P301S Cgas* +/*+* vs *P301S Cgas−/−* ** p = 0.0028. One-way ANOVA followed by Tukey’s multiple comparisons test for each gene. **d**. Heatmap summary of genes with lower induction in tau-treated *Cgas*^*−/−*^, compared *Cgas*^*+/+*^ microglia. **e**. BV2 cells stably expressing IfnB-responsive luciferase reporter construct show dose-dependent increase of secreted luciferase activity in response to cGAMP. Data are reported as mean ± SEM. n = 4 biologically independent samples. Adjusted p-value <0.0001 for 0 vs 2.5, 0 vs 5, 0 vs 10, 0 vs 20. One-way ANOVA followed by Dunnett multiple comparison test. **f**. Quantification of luminescence intensity in BV2 IfnB luciferase reporter cells treated or not with HT-DNA or tau fibrils. Data are reported as mean ± SEM. n = 3 biologically independent samples. **** p < 0.0001. Two-tailed unpaired t-test. **g**. Control and mtDNA-depleted (ρ ͦ) IfnB luciferase-reporter BV2 cells were stimulated or not with ABT-737 + QVD (10 μm each). IfnB signal and viability were measured 18 h later. IfnB-luciferase signal is shown normalized to Cell TiterGlo signal to correct for viability/cell count. Data are reported as mean ± SEM. n = 3 biologically independent samples. *** p = 0.0004, **** p < 0.0001. Two-way ANOVA. **h**. Immunostaining for γH2A.X in primary cultured mouse microglia treated with tau fibrils. **i**. Quantification of the percentage of γH2A.X + cells in (h). Data are reported as mean ± SEM. n = 4 biologically independent samples. ** p < 0.0029. Two-tailed unpaired t-test. **j**. Visualization of extranuclear DNA foci indicated by arrows using DAPI staining. **k**. Quantification of the percentage of cells with extranuclear DNA foci in (j). Data are reported as mean ± SEM. n = 4 biologically independent samples. ns: not significant. Two-tailed unpaired t-test.

We next sought to determine if pathogenic tau activates IFN signaling in microglia. Using enzyme-linked immunosorbent assays (ELISAs) and MAGPIX assays, we measured IFNβ, CXCL10 and CCL5 protein expression in culture medium from primary microglia treated with tau fibrils (Fig. 2a). Treatment with fibrils led to robust TBK1 phosphorylation, suggesting activation of cGAS–STING signaling (Fig. 2b,c). Genetic ablation of *Cgas* in primary microglia abolished tau-mediated cytokine expression (Fig. 2d). The responses of *Cgas*^−/−^ and *Cgas*^+/+^ primary microglia treated with tau fibrils or herring testis DNA (HT-DNA) were compared using RNA-seq. Almost 80% of genes upregulated by tau were upregulated by HT-DNA, and IFN signaling pathways were overrepresented in these shared DEGs (Fig. 2e,f). Consistent with the canonical role of cGAS as a cytoplasmic DNA sensor, microglial transcriptional responses to HT-DNA were abolished in *Cgas*^−/−^ microglia (Extended Data Fig. 2a,b). *Cgas* deletion reduced the expression of tau-induced IFN-stimulated genes (*Stat1*, *Ddx60*, *Isg20*, *Rnf213*, *Parp12*, *Ifi35*, *Sp100* and others; Fig. 2g) and genes encoding cytokines and inflammatory cytokines (Extended Data Fig. 2c,d).

How does tau induce cGAS-mediated IFN activation? In primary microglia treated with tau, electron microscopy and immunogold labeling showed that tau localized to lysosomes and mitochondria (Fig. 2h). Tau fibril treatment also led to mitochondrial DNA (mtDNA) release into the cytosol (Fig. 2i). To quantify IFN responses, we generated an IFNβ–luciferase reporter BV2 cell line, which upregulates IFNβ–luciferase in response to cGAS agonists (cGAMP and HT-DNA) and tau fibrils (Extended Data Fig. 2e,f). We generated mtDNA-depleted cells by treating IFNβ reporter BV2 cells with dideoxycytidine (ddC) or low-dose ethidium bromide (EtBr)^20,21^. mtDNA was depleted in a dose-dependent manner after ddC and EtBr treatment (Fig. 2j). In control experiments, the BCL-2 inhibitor ABT-737 and caspase inhibitor Q-VD-OPH (QVD), which trigger mtDNA leakage without apoptosis induction^22,23^, activated IFNβ-dependent luciferase expression that was dampened with ddC- or EtBr-induced mtDNA depletion, confirming activation of IFN-I by cytosolic mtDNA (Extended Data Fig. 2g). After tau treatment, ddC and EtBr dampened the microglial IFNβ response in a dose-dependent manner, implicating mtDNA in tau-dependent IFN responses in microglia (Fig. 2k). cGAS can also be activated by cytoplasmic DNA from the nucleus, such as cytoplasmic chromatin fragments (CCF)^24^. While tau fibrils increased nuclear DNA damage in cultured primary microglia, they did not induce accumulation of CCF (Extended Data Fig. 2h–k).

**Extended Data Fig. 3:**
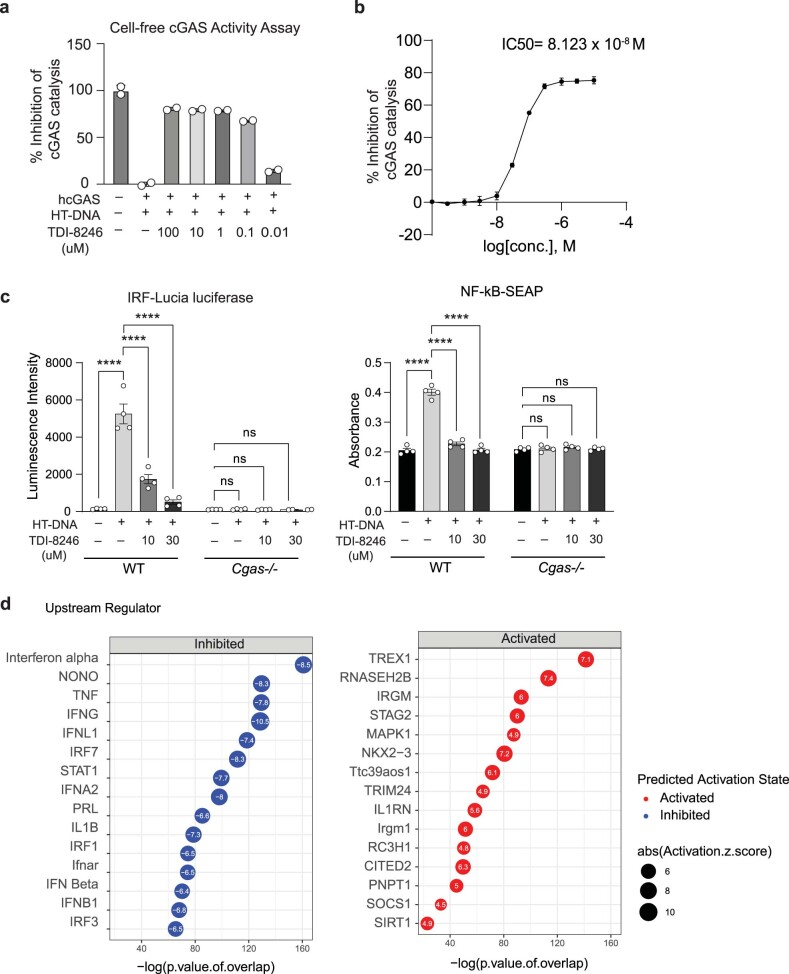
Activity and specificity of a human cGAS inhibitor TDI-8246 (Related to Fig. 2). **a**. Inhibitory effects of TDI-8246 (0.01–100 µM concentration) on human cGAS/HT-DNA-catalyzed conversion of ATP and GTP to produce cGAMP measured by remaining ATP concentrations in the reactions using ATP Glo assay. **b**. Dose-dependent inhibition of cGAS activity (as measured in a) in response to HT-DNA. IC_50_ = 8.123 × 10^−8^ M. Data are reported as mean ± SD. n = 3 biologically independent samples per condition. **c**. Quantification of IRF-Lucia luciferase luminescence intensity and NF-κB-SEAP absorbance of WT and *Cgas−/−* THP-1 dual cells treated with HT-DNA and TDI-8246 as indicated. Data are reported as mean ± SEM. n = 4 biologically independent samples. **** p < 0.0001. Two-way ANOVA followed by Tukey’s multiple comparisons test (n = 4). **d**. Predicted upstream regulators associated with DEGs in DMSO + tau vs TDI-8246+tau from ingenuity pathway analysis. *P*-value overlap < 0.05, Activation Z score > 2 or < -2. Right-tailed Fisher’s exact test.

To investigate cGAS activation in human microglia, we next differentiated human induced pluripotent stem cells (iPSCs) into microglial-like cells (hMGs) followed by tau treatment. As in mouse microglia, tau particles were detected in the mitochondria of fibril-treated hMGs (Fig. 2l). Tau stimulation in hMGs induced phosphorylation of STING at Ser 366 (Fig. 2m). To determine the role of cGAS–STING signaling in response to tau in human microglia, we synthesized a human cGAS inhibitor TDI-8246 (ref. ^25^). Using cell-free and cell-based assays, we established the effective dose and specificity of TDI-8246 in response to HT-DNA (Extended Data Fig. 3a–c). Inhibition of cGAS with TDI-8246 or STING with inhibitor H-151 blocked STING phosphorylation (Fig. 2n) and IFN-inducible cytokines CXCL10 and CCL5 in response to tau (Fig. 2o). RNA-seq analyses of hMGs treated with tau and TDI-8246 showed that cGAS inhibition reversed 554 of 3,337 genes upregulated by tau (Fig. 2p and Supplementary Table 4), which were highly enriched for IFN signaling (Fig. 2q). IFNα and other IFN-related TFs were the top upstream regulators reversed by the cGAS inhibitor TDI-8246 (Extended Data Fig. 3d).

### cGAS loss mitigates tauopathy-induced microglial IFN-I in vivo

To assess cGAS in tauopathy in vivo, we crossed *P301S* transgenic mice with *Cgas*^–/–^ mice to generate transgenic and non-transgenic litters expressing two, one or no copies of functional *Cgas*. We performed snRNA-seq of hippocampi from 8- to 9-month-old *Cgas*^+/+^, *P301S* *Cgas*^+/+^, *P301S Cgas*^+/–^ and *P301S Cgas*^–/–^ mice. Stringent quality control excluded sequencing reads from multiplets by using DoubletFinder^26^ and low-quality nuclei by thresholding gene counts, unique molecular identifier counts and percentage of mitochondrial genes per nuclei. Unsupervised clustering grouped 221,150 high-quality nuclei into transcriptionally distinct clusters that represented all major brain cell types (Supplementary Fig. 1).

**Fig. 3:**
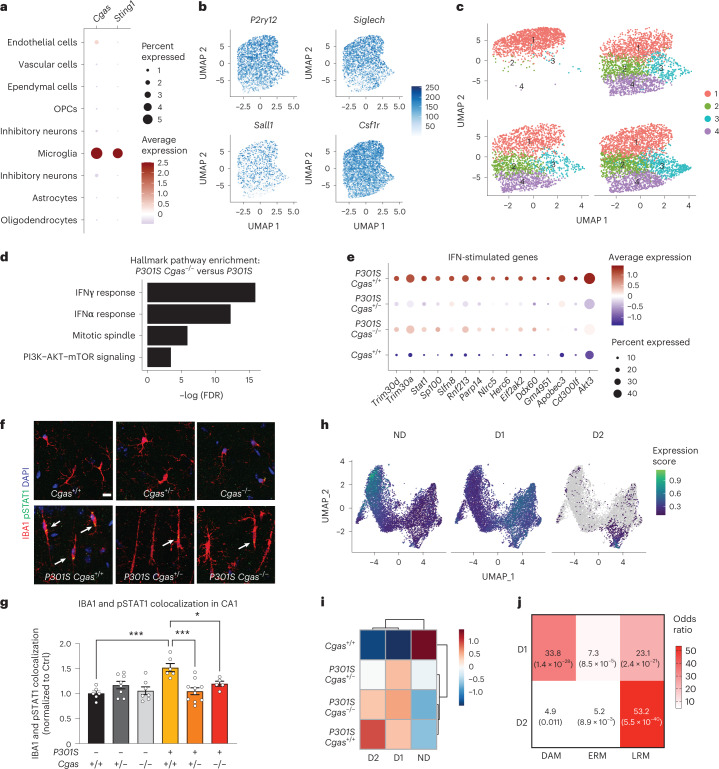
cGAS promotes a tauopathy-associated microglial IFN signature distinct from DAMs. **a**, Dot plot of normalized cell-type expression of *Cgas* and *Sting1* (*Tmem173*) in snRNA-seq samples; OPCs, oligodendrocyte progenitor cells. **b**, Uniform manifold approximation and projection (UMAP) plots showing strong expression of marker genes *P2ry12*, *Siglech*, *Sall1* and *Csf1r* in snRNA-seq microglial populations (*n* = 6 per genotype except for *n* = 5 for *Cgas*^+/+^). **c**, UMAP plots colored according to microglial subclusters and split by genotype. **d**, Gene set enrichment analysis showing that hallmark pathways associated with significantly downregulated genes (log_2_ FC < −0.1 and FDR < 0.05) in *P301S Cgas*^−/−^ versus *P301S* are IFN pathways. **e**, Dot plot showing IFN-stimulated genes that are significantly lower in *P301S Cgas*^+/*−*^ and *P301S Cgas*^−/−^ microglia than in *P301S Cgas*^+/+^ microglia. **f**, Representative ×63 confocal images of immunostaining of pSTAT1 in the CA1 stratum radiatum of the mouse hippocampus; scale bar, 10 μm. **g**, Mean intensity of pSTAT1 measured in IBA1^+^ microglia in the CA1 striatum radiatum. Each circle represents the average intensity measurement of three images per animal. Data are reported as mean ± s.e.m.; *n* = 6 *Cgas*^+/+^, *n* = 8 *Cgas*^+/−^, *n* = 7 *Cgas*^−/−^, *n* = 6 *P301S Cgas*^+/+^, *n* = 9 *P301S Cgas*^+/−^, *n* = 5 *P301S Cgas*^−/−^. *Cgas*^+/+^ versus *P301S Cgas*^+/+^: ***P = 0.0002, *P301S Cgas*^+/+^ versus *P301S Cgas*^+/−^: ***P = 0.0002, *P301S Cgas*^+/+^ versus *P301S Cgas*^−/−^: *P = 0.0415. Data were analyzed by two-way ANOVA followed by a Tukey multiple comparison test. **h**, UMAP plots showing gene expression modules associated with microglial transformation; ND, non-disease. **i**, Heat map showing the association of gene modules with genotype. **j**, Analysis of D1 and D2 markers compared to DAM, early-response microglia (ERM) signatures and late-response microglia (LRM) signatures.

**Extended Data Fig. 4:**
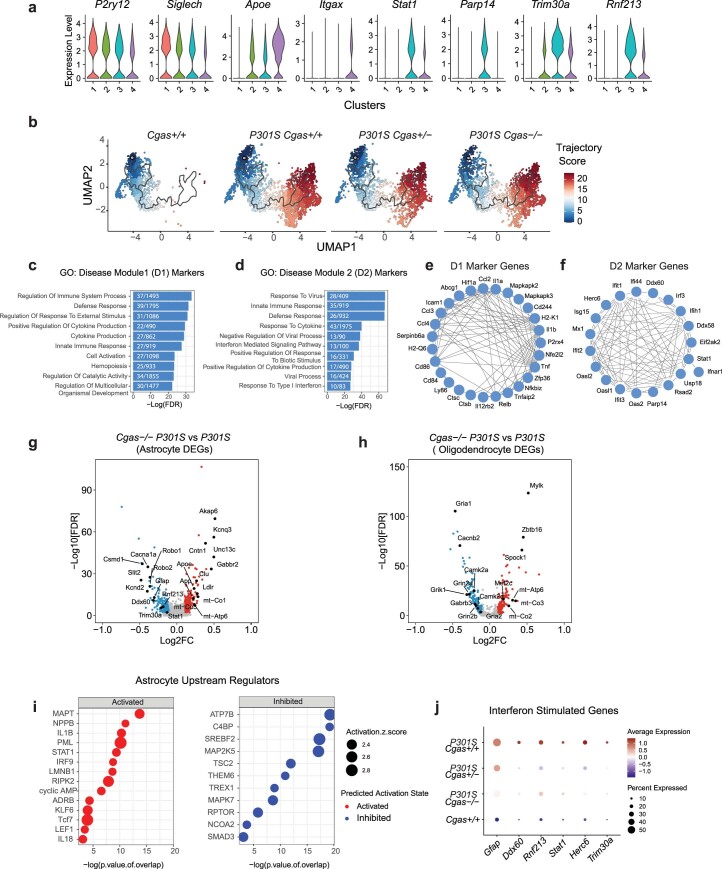
snRNA-seq analyses of microglia, astrocytes, and oligodendrocytes (Related to Fig. 3). **a**. Violin plots showing expression level of homeostatic (*P2ry12, Siglech)*, disease-associated (*Apoe, Itgax*) and interferon (*Parp14, Stat1, Trim30a, Rnf213*) genes in microglia clusters. **b**. Microglial trajectory plots colored by cell state trajectory scores identified using Monocle 3 and split by genotype. **c**. Top gene ontology terms enriched in disease module 1 markers. **d**. Top gene ontology terms enriched in disease module 2 markers. **e**. String interaction plot of disease module 1 markers. **f**. String interaction plot of disease module 2 markers. **g**. Volcano plot showing representative DEGs in *P301S Cgas*^*−/−*^, compared to *P301S Cgas*^*+/+*^ astrocytes. (logFC > =0.1 or < =-0.1, FDR < 0.05). **h**. Volcano plot showing representative DEGs in *P301S Cgas*^*−/−*^, compared to *P301S Cgas*^*+/+*^ oligodendrocytes. (logFC > = 0.1 or <=−0.1, FDR < 0.05). **i**. Predicted upstream regulators associated with DEGs in *P301S Cgas*^*−/−*^, compared to *P301S Cgas*^*+/+*^ astrocytes, from Ingenuity Pathway Analysis. *P*-value overlap < 0.05, Activation Z score > 2 or < -2. Right-tailed Fisher’s exact test. **j**. Dot plot showing interferon-stimulated genes that are significantly lower in *P301S Cgas*^*+/−*^ and *P301S Cgas*^*−/−*^ astrocytes than in *P301S Cgas*^*+/+*^ astrocytes.

In our snRNA-seq data, *Cgas* and *Sting1* (*Tmem173*) were detected mainly in the microglial cluster characterized by the expression of microglial markers (*Csf1r*, *P2ry12* and *Siglech*; Fig. 3a,b). To determine how *Cgas* reduction affects microglial responses to tauopathy, microglia were subclustered into four subpopulations. *Cgas*^+/+^ samples included primarily cluster 1 microglia, but all clusters were found in tauopathy samples, confirming robust transformation of microglial states in tauopathy^27^ (Fig. 3c). As expected, cluster 1 expressed high levels of homeostatic genes, *P2ry12* and *Siglech*, and clusters 2, 3 and 4 showed reduced expression of these homeostatic genes and simultaneous upregulation of disease-associated (*Apoe* and *Itgax*) and IFN-stimulated genes (*Stat1*, *Parp14*, *Trim30a* and *Rnf213*). Cluster 3 was enriched with IFN genes specifically, whereas cluster 4 was enriched with the disease-associated microglial (DAM) genes reported in a mouse amyloid model^28^ (Extended Data Fig. 4a and Supplementary Table 5). DEG analysis between *P301S Cgas*^+/+^ and *P301S Cgas*^−/−^ microglia revealed ‘IFNγ’ and ‘IFNα response’ as the top pathways suppressed by *Cgas* deletion (Fig. 3d). Expression of IFN-stimulated genes (*Trim30a*, *Trim30d*, *Stat1*, *Ddx60*, *Rnf213*, *Parp14* and others) was stronger in microglia of *P301S Cgas*^+/+^ mice and was suppressed in *P301S Cgas*^+/−^ and *P301S Cgas*^−/−^ microglia (Fig. 3e and Supplementary Table 5). To validate suppression of IFN responses in microglia by *Cgas* reduction, we performed immunofluorescent labeling of phosphorylated STAT1 (pSTAT1). Microglia in *P301S* transgenic mice with tauopathy were elongated with increased pSTAT1 signals. The increased pSTAT1 signal in microglia of the hippocampal CA1 region of *P301S Cgas*^+/+^ brains was diminished in *P301S Cgas*^+/−^ and *P301S Cgas*^−/−^ brains (Fig. 3f,g).

We next used trajectory analysis to model cGAS in microglial transformation from homeostatic to disease states (Extended Data Fig. 4b)^29^ and identified three gene modules (Fig. 3h). Gene ontology analysis identified overrepresented gene signatures associated with the microglial disease modules. Disease module 1 (D1) markers were associated with cell activation, defense response and immune system, and disease module 2 (D2) markers were enriched with genes involved in response to virus and IFN-I (Extended Data Fig. 4c,d and Supplementary Table 5). D1 genes included DAM genes (*Ctsb*, *H2-K1* and *Hif1a)*. D2 included IFN genes (Extended Data Fig. 4e,f). Compared to *P301S Cgas*^+/+^, *P301S Cgas*^+/−^ and *P301S Cgas*^−/−^ microglia exhibited no change in D1 but less D2 (Fig. 3i). D2 correlated best with a late-response microglial signature associated with synapse and neuron loss and cognitive impairment in a p25 induction model of neurodegeneration (Fig. 3j)^30^.

Consistent with the strongest expression of *Cgas* in microglia, ablation of *Cgas* modestly downregulated IFN-stimulated genes in astrocytes but not in oligodendrocytes (Extended Data Fig. 4g–j and Supplementary Table 6).

### cGAS loss rescues memory, synapse loss and plasticity

**Fig. 4:**
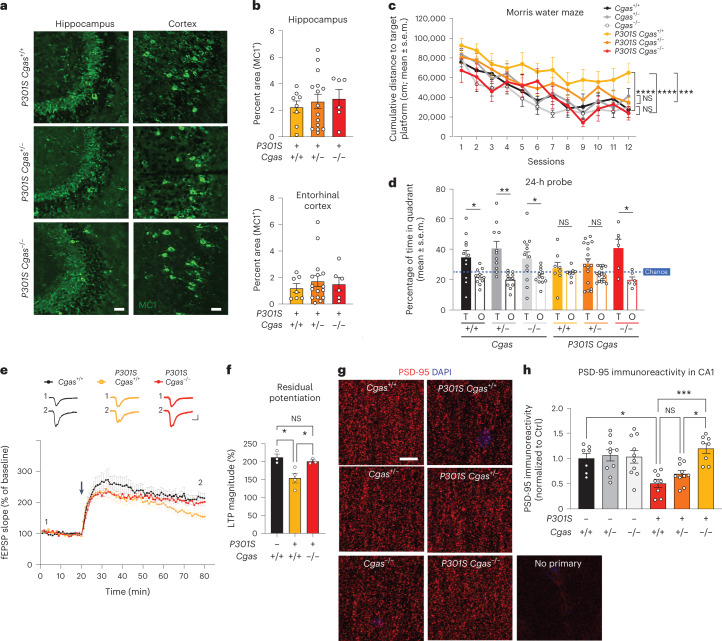
Loss of cGAS rescues tauopathy-induced hippocampal synapse loss, synaptic plasticity and memory deficits without affecting tau load. **a**, Representative immunofluorescence images of MC1 immunostaining in the hippocampi and entorhinal cortexes of 8- to 9-month-old mice; scale bar, 50 µm. **b**, Percentage of MC1^+^ area in the hippocampus or entorhinal cortex of *P301S Cgas*^+/+^, *P301S Cgas*^+/−^ and *P301S Cgas*^−/−^ mice. Data were analyzed by two-way ANOVA. **c**, Cumulative search distance to target platform during hidden trials (sessions 1**–**12) in a Morris water maze assessment of spatial learning and memory in 7- to 8-month-old *P301S Cgas*^+/+^, *P301S* *Cgas*^+/−^ and *P301S Cgas*^−/−^ mice and their non-transgenic littermates. Males and females were tested on separate days. Data represent both sexes combined; *n* = 12 *Cgas*^+/+^, *n* = 11 *Cgas*^+/*−*^, *n* = 11 *Cgas*^−/−^, *n* = 8 *P301S Cgas*^+/+^, n = 17 *P301S Cgas*^+/−^, *n* = 6 *P301S Cgas*^−/−^. *Cgas*^+/+^ versus *P301S Cgas*^+/+^: ****P < 0.0001; *P301S Cgas*^+/+^ versus *P301S Cgas*^−/−^: ****P < 0.0001; *P301S Cgas*^+/+^ versus *P301S Cgas*^+/−^: ***P = 0.0002. Data were analyzed by two-way ANOVA with a Tukey multiple comparisons test; NS, not significant. **d**, Percentage of time spent in the target (T) or the average time spent in the nontarget (others; O) quadrants during the 24-h probe in the W Morris water maze. assessment; *n* = 12 *Cgas*^+/+^, *P = 0.0293; *n* = 11 *Cgas*^+/−^, ** *P =* 0.0035; *n* = 11 *Cgas*^−/−^, *P = 0.0370; *n* = 8 *P301S Cgas*^+/+^; *n* = 17 *P301S Cgas*^+/−^; *n* = 6 *P301S Cgas*^−/−^, *P = 0.0209. Data were analyzed by one-tailed paired *t*-test. **e**, Field excitatory postsynaptic potentials (fEPSPs) were recorded in the dentate gyrus molecular layer, and a TBS protocol was applied (arrow) to the perforant pathway to induce LTP. Representative traces show fEPSPs before and after LTP induction (top); scale bars, 0.4 mV and 5 ms. The fEPSP slope measurements made up to 60 min after TBS were normalized to the mean baseline fEPSP slope before LTP induction (bottom, 8–11 slices from three to four mice: *n* = 3 *Cgas*^+/+^, *n* = 4 *P301S Cgas*^+/+^, *n* = 3 *P301S Cgas*^−/−^). **f**, The LTP magnitude was calculated from the normalized mean fEPSP slope 55–60 min after TBS was applied. Data are reported as mean ± s.e.m. (8–11 slices from three to four mice per group; *n* = 3 *Cgas*^+/+^, *n* = 4 *P301S Cgas*^+/+^, *n* = 3 *P301S Cgas*^−/−^. *Cgas*^+/+^ versus *P301S Cgas*^+/+^: *P = 0.0191; *P301S Cgas*^+/+^ versus *P301S Cgas*^−/−^: *P = 0.0477. Data were analyzed by one-way ANOVA followed by a Tukey multiple comparisons test. **g**, Representative confocal images of the hippocampal CA1 striatum radiatum labeled with PSD-95 antibody; scale bar, 10 μm. **h**, Mean intensity of PSD-95 puncta measured in the CA1 striatum radiatum. Each circle represents the average intensity measurement of three to five images per animal. Data are presented as normalized to control. Error bars represent mean ± s.e.m. (*n* = 7 *Cgas*^+/+^, *n* = 10 *Cgas*^+/−^, *n* = 10 *Cgas*^−/−^, *n* = 8 *P301S Cgas*^+/+^, *n* = 10 *P301S Cgas*^+/−^, *n* = 8 *P301S Cgas*^−/−^; *Cgas*^+/+^ versus *P301S Cgas*^+/+^: *P = 0.0275; *P301S Cgas*^+/−^ versus *P301S Cgas*^−/−^: *P = 0.0111; *P301S Cgas*^+/+^ versus *P301S Cgas*^−/−^: ***P = 0.0004). Data were analyzed by two-way ANOVA followed by a Tukey multiple comparisons test.

**Extended Data Fig. 5:**
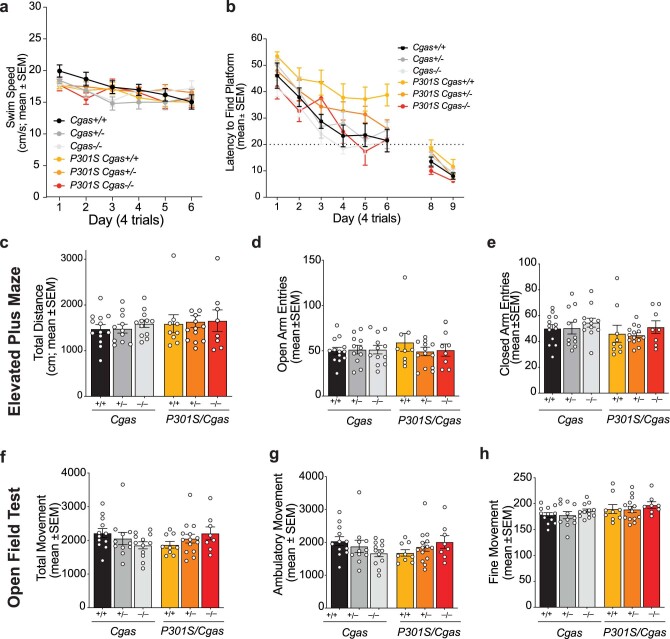
Loss of *Cgas* does not affect swim speed, overall activity, or anxiety in tauopathy mice (Related to Fig. 4). **a**. Average swim speeds measured during each date of hidden platform trials (days 1–6). Data are reported as mean ± SEM. n = 12 *Cgas* +*/+*, n = 11 *Cgas* +/−, n = 11 *Cgas−/−*, n = 8 *P301S Cgas* +*/+*, n = 17 *P301S Cgas* +/−, n = 6 *P301S Cgas−/−*. **b**. Cued platform probes (days 8–9) showing lack of visual impairment in all mice tested. Data are reported as mean ± SEM. n = 12 *Cgas* +*/+*, n = 11 *Cgas* +/−, n = 11 *Cgas−/−*, n = 8 *P301S Cgas* +*/+*, n = 17 *P301S Cgas* +/−, n = 6 *P301S Cgas−/−*. **c**–**e**. Elevated plus maze assessment of anxiety levels by measuring total distance traveled, open arm entries and closed-arm entries. Data are reported as mean ± SEM. n = 13 *Cgas* +*/+*, n = 11 *Cgas* +/−, n = 12 *Cgas−/−*, n = 9 *P301S Cgas* +*/+*, n = 14 *P301S Cgas* +/−, n = 8 *P301S Cgas−/−*. No genotype effects observed for all groups. Two-way ANOVA. **f**–**h**. Open-field assessment of total, fine and ambulatory movement showed no genotype effects of overall activity in 8–9-month-old mice. Data are reported as mean ± SEM. n = 12 *Cgas* +*/+*, n = 11 *Cgas* +/−, n = 12 *Cgas−/−*, n = 9 *P301S Cgas* +*/+*, n = 14 *P301S Cgas* +*/*−, n = 8 *P301S Cgas−/−*. Two-way ANOVA.

We examined tau load with a conformation-specific tau antibody, MC1, and observed no differences in tau aggregates in hippocampi and entorhinal cortices of transgenic mice (Fig. 4a,b). To determine if *Cgas* deletion affected tauopathy-induced spatial learning and memory, we used the Morris water maze test. Spatial learning was assessed in hidden platform trials over six consecutive days by measuring the distance to locate the platform in each trial (Fig. 4c). *Cgas*^+/–^ and *Cgas*^–/–^ mice phenocopied *Cgas*^+/+^ mice, but *P301S Cgas*^+/+^ mice exhibited impaired learning compared to non-transgenic mice. Despite similar tau loads, *P301S Cgas*^+/−^ and *P301S Cgas*^−/−^ mice exhibited stronger spatial learning than *P301S Cgas*^+/+^ mice in the hidden platform trial. In the 24-h probe trials to assess spatial memory, *P301S Cgas*^−/−^ mice spent more time exploring the target quadrant than other quadrants. *P301S Cgas*^+/+^ mice could not discriminate the target from others (Fig. 4d). Swim speeds, vision, overall activity and anxiety were unaltered across all genotypes (Extended Data Fig. 5a–h).

Tau-induced deficits in hippocampal synaptic plasticity are linked to tauopathy-related memory loss^31^. Theta burst stimulation (TBS) induced long-term potentiation (LTP) similarly in *P301S Cgas*^+/+^ and *P301S Cgas*^−/−^ slices at early-phase LTP. However, LTP magnitude was less in *P301S Cgas*^+/+^ than in *P301S Cgas*^−/−^ slices by 60 min after induction, indicating that late-phase LTP impairment in *P301S Cgas*^+/+^ hippocampi was rescued by *Cgas* deletion (Fig. 4e,f). Quantification of densities of PSD-95, a marker of excitatory postsynaptic terminals, in the striatum radiatum revealed that cGAS ablation rescued tauopathy-induced PSD-95 loss in CA1 pyramidal neurons (Fig. 4g,h).

### Neuronal MEF2C transcriptional network enhanced by *Cgas* loss

**Extended Data Fig. 6:**
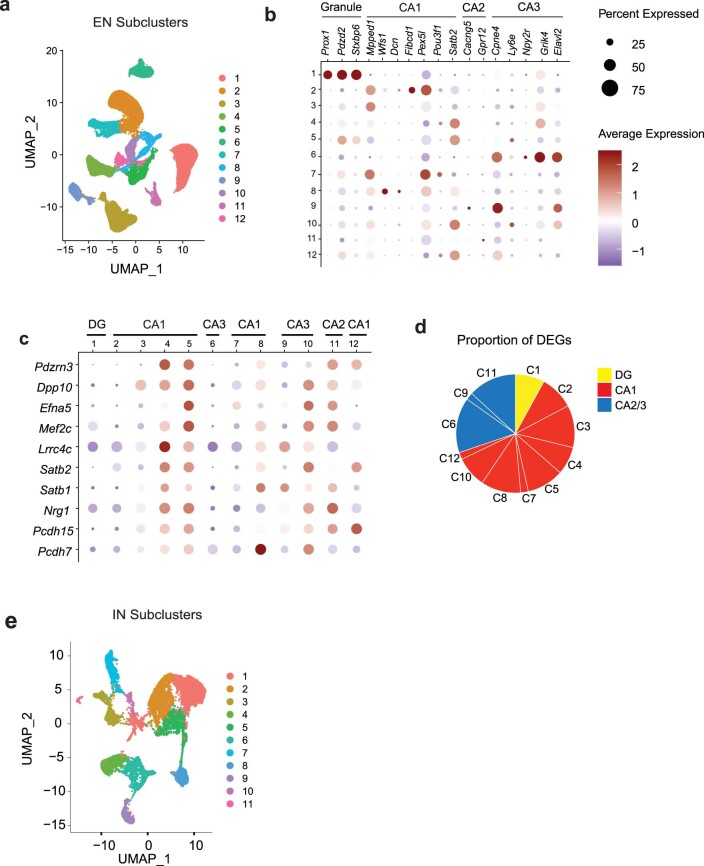
Cellular markers of excitatory and inhibitory neurons identified in snRNA-seq analyses of hippocampi of *P301S* mice with or without cGAS (Related to Fig. 5). **a**. UMAP projection showing 12 distinct hippocampal excitatory neuron populations identified using unsupervised clustering of snRNA-seq data. **b**. Dot plot showing classification of excitatory neuron clusters by expression of granule, CA1, CA2, and CA3 markers. **c**. Dot plot of top shared DEGs from excitatory neuron clusters showing their enrichment in the different excitatory neuron clusters categorized according to granule, CA1, and CA2/3 markers. **d**. Pie chart summarizing the proportion of DEGs from clusters pertaining to dentate gyrus (DG), CA1, and CA2/3 clusters. **e**. UMAP projection showing 11 distinct hippocampal inhibitory neuron populations identified using unsupervised clustering of snRNA-seq data.

To interrogate how *Cgas* loss protects against tau pathology in the hippocampal circuit, we performed subclustering analyses of excitatory neuron (EN) populations and identified 12 transcriptionally distinct subpopulations that showed non-overlapping expression of dentate granule, CA1 and CA2/CA3 neuron-specific subtype markers^32–35^ (Extended Data Fig. 6a,b and Supplementary Table 7). DEGs from the comparison of ENs of *P301S Cgas*^−/−^ and *P301S Cgas*^+/+^ were enriched in clusters expressing CA1 and CA3 to lesser extent but not those expressing granule cell markers (Extended Data Fig. 6c,d), consistent with our observation of the protective effects of *Cgas* deletion on CA1 synaptic integrity.

**Fig. 5:**
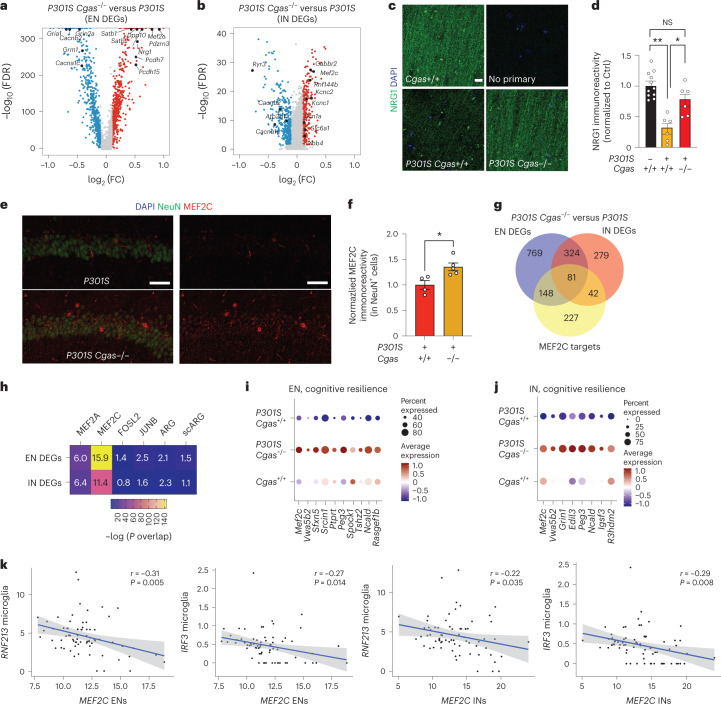
The MEF2C transcription network is enhanced by *Cgas* deletion and inversely correlates with the microglial IFN response in mice and humans. **a**, Volcano plot showing representative DEGs that are upregulated in *P301S Cgas*^−/−^ compared to in *P301S Cgas*^+/+^ ENs; log_2_ FC of >0.1 or <−0.1, FDR < 0.05. **b**, Volcano plot showing representative DEGs that are upregulated in *P301S Cgas*^−/−^ compared to in *P301S Cgas*^+/+^ INs; log_2_ FC of >0.1 or <−0.1, FDR < 0.05. **c**, Representative confocal images of immunostaining of NRG1 in the CA1 stratum radiatum of the mouse hippocampus; scale bar, 10 μm. **d**, Mean intensity of NRG1 measured in the CA1 striatum radiatum. Each circle represents the average intensity measurement of three images per animal. Data are reported as mean ± s.e.m.; *n* = 11 *Cgas*^+/+^, *n* = 6 *P301S Cgas*^+/+^, *n* = 8 *P301S Cgas*^+/−^, *n* = 6 *P301S Cgas*^−/−^. *Cgas*^+/+^ versus *P301S Cgas*^+/+^: **P = 0.0016, *P301S Cgas*^+/+^ versus *P301S Cgas*^−/−^*:* *P = 0.0203. Data were analyzed by two-way ANOVA followed by a Tukey multiple comparisons test. **e**, Representative ×40 confocal images of immunostaining of MEF2C and NeuN in the CA1 pyramidal layer of the mouse hippocampus; scale bar, 50 μm. **f**, Mean intensity of MEF2C in MEF2C^+^NeuN^+^ neurons in the CA1 pyramidal layer. Each circle represents the average intensity measurement of three images per animal. Data are presented as normalized to control. Data are reported as mean ± s.e.m.; *n* = 4 *P301S Cgas*^+/+^, *n* = 5 *P301S Cgas*^−/−^; *P = 0.0178. Data were analyzed by two-tailed unpaired *t*-test **g**, Venn diagram of the overlaps among EN DEGs, IN DEGs and MEF2C target genes. **h**, Heatmap showing the overlap between EN/IN DEGs and lists of TF target genes (MEF2A, MEF2C, FOSL2 and JUNB) and activity-induced DEGs (ARG and scARG). Numbers in each box represent the overlapping odds ratio. Overlapping *P* values were calculated with a Fisher’s exact test. **i**, Dot plot showing the expression of DEGs significantly upregulated by *Cgas* deletion (FDR < 0.05, log_2_ FC ≥ 0.1) that are positively correlated with human cognitive resilience in EN clusters. **j**, Dot plot showing the expression of significantly upregulated DEGs by *Cgas* deletion (FDR < 0.05, log_2_ FC ≥ 0.1) that are positively correlated with human cognitive resilience in IN clusters. **k**, Simple linear regression analysis with standard error showing negative correlations between the expression of *MEF2C* in ENs and *RNF213* (*r* = −0.31, *P* = 0.005) and *IRF3* (*r* = −0.27, *P* = 0.014) in microglia and *MEF2C* in INs and *RNF213* (*r* = −0.22, *P* = 0.035) and *IRF3* (*r* = −0.29, *P* = 0.008) in microglia in 70 individuals with AD.

Top DEGs in ENs included genes in transcription regulation and chromatin remodeling (*Mef2c*, *Satb1* and *Satb2*), excitability (a potassium channel regulator, *Dpp10*) and synapse maintenance (*Nrg1*, *Pcdh7* and *Pcdh15* (refs. ^36–39^); Fig. 5a and Supplementary Table 7). Subclustering of pan-interneuron marker GAD1^+^ and GAD2^+^ neuron populations identified 11 inhibitory neuron (IN) subpopulations (Extended Data Fig. 6e)^32,35^. Genes upregulated by *Cgas* deletion in interneurons included those in GABAergic signaling, a GABA transporter (*Slc6a1*), a GABA receptor (*Gabbr2*) and ion channels regulating neuronal excitability and firing (*Kcnc1* and *Kcnc2*). Sodium channel Nav1.1 (*Scn1a*), which is downregulated in parvalbumin interneurons of human AD brains and is implicated in hyperexcitability and cognitive deficits in an AD mouse model^40^, was upregulated by *Cgas* deletion. Genes downregulated by *Cgas* deletion in INs included calcium channels (*Cacnb2* and *Cacna1e*) and ryanodine receptor calcium release channel (*Ryr3*; Fig. 5b and Supplementary Table 7). Immunofluorescent labeling of NRG1 in the CA1 validated downregulation of NRG1 protein in tauopathy and its rescue by deleting *Cgas* (Fig. 5c,d).

**Extended Data Fig. 7:**
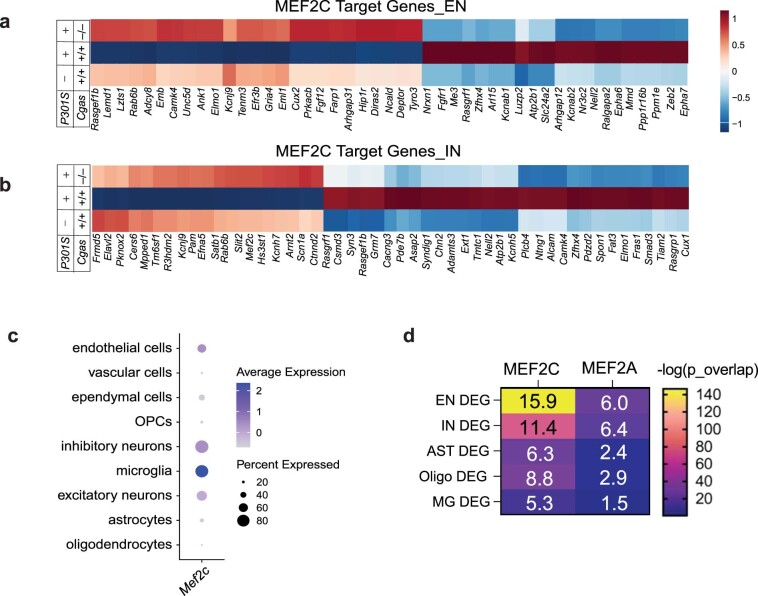
Effects of *Cgas* deletion on MEF2C transcriptional network in different cell types (Related to Fig. 5). **a**. Heatmap of the expression of significant DEGs (FDR < 0.05, logFC > =0.1 or < =-0.1) that are MEF2C targets in WT, *P301S*, and *P301S Cgas−/−* excitatory neuron clusters. **b**. Heatmap of the expression of significant DEGs (FDR < 0.05, logFC > =0.1 or < =-0.1) that are MEF2C targets in WT, *P301S*, and *P301S Cgas−/−* inhibitory neuron clusters. **c**. Dot plot showing normalized cell-type expression of *Mef2c* RNA in snRNA-Seq samples. **d**. Heatmap showing the overlap between DEGs and lists of MEF2 transcription factor target genes (MEF2A and MEF2C) across different cell types. Numbers in each box represents the overlapping odds ratio. *P* value calculated with Fisher’s exact test.

Among the top DEGs upregulated in ENs and INs in *P301S Cgas*^−/−^ mice was *Mef2c*, a TF implicated in late-onset AD^41^ and linked to cognitive resilience in AD brains^16^ (Fig. 5a,b). We confirmed elevated MEF2C expression in the hippocampi of *P301S Cgas*^−/−^ mice (Fig. 5e,f). Comparing the DEGs (*P301S Cgas*^−/−^ versus *P301S*) in ENs and INs with MEF2C target genes^16^ revealed a substantial overlap (Fig. 5g) and highly significant overrepresentation (Fig. 5h) of MEF2C target genes in *P301S Cgas*^−/−^ ENs and INs. These neuronal transcriptomic changes were specific to the MEF2C network but not other MEF2 family members or TFs regulating neuronal activity, as we observed no strong overlap among the DEGs and MEF2A, FOSL2 and JUNB target genes and two reports of transcriptional changes after induced neuronal activity^16^ (Fig. 5h). Indeed, *Cgas* loss induced specific enhancement of the MEF2C transcriptional network in *P301S* mice as a transcription activator and a repressor in ENs and INs. In ENs, MEF2C target genes rescued by *Cgas* deletion in *P301S* mice included genes involved in axonal guidance, dendritic growth and synaptic maintenance (*Tenm3*, *Unc5d*, *Nrxn1*, *Lzts1* and *Gria4*) and those in regulating calcium signaling/homeostasis (*Cacng3*, *Ncald* and *Slc24a3*; Extended Data Fig. 7a). Similarly, *Cgas* deletion in INs also rescued MEF2C target genes involved in axonal guidance, growth and synaptic maintenance (*Elavl2*, *Slit2* and *Ctnnd2*) and those regulating calcium signaling/homeostasis (*Cacng3* and *Camk4*; Extended Data Fig. 7b). In agreement with the finding that MEF2C overexpression ameliorated hyperexcitability in *P301S* mice^16^, *Cgas* deletion rescued genes in regulating neuronal excitability, such as potassium channel and regulatory subunits *Kcnab2* and *Dpp10* in ENs and *Kcnj9* and *Kcnip2* in INs. Among the MEF2C target genes downregulated by *Cgas* deletion in ENs and INs were genes involved in Eph/Ephrin signaling (*Eph6* and *Eph7*), the blockade of which can promote regeneration during injury^42^ (Extended Data Fig. 7a,b and Supplementary Table 7). MEF2C target genes upregulated in *Cgas*^–/–^ ENs and INs overlap with genes in cognitive resilience in AD brains, including *Ncald*, *Rasgef1b*, *Igsf3* and *R3hdm2* (Fig. 5i,j). Thus, the MEF2C transcription network could drive the protective mechanism underlying *Cgas*^–/–^ neurons in tau pathology.

While *Mef2c* is highly expressed in microglia (Extended Data Fig. 7c), microglial *Mef2c* expression was not changed, and microglial DEGs showed the lowest enrichment for MEF2C targets, whereas the highest enrichment for MEF2C targets was in DEGs of ENs and INs (Extended Data Fig. 7d).

### Microglial IFN activation suppresses neuronal MEF2C transcription

We next investigated the relationship between microglial IFN response and neuronal MEF2C in individuals with AD in two published datasets of single-nuclei transcriptomics with a combined 70 individuals^27,43^. Microglial expression of IFN-inducible genes *RNF213* and *IRF3* was inversely correlated with *MEF2C* expression in ENs and INs (Fig. 5k and Supplementary Table 8).

**Extended Data Fig. 8:**
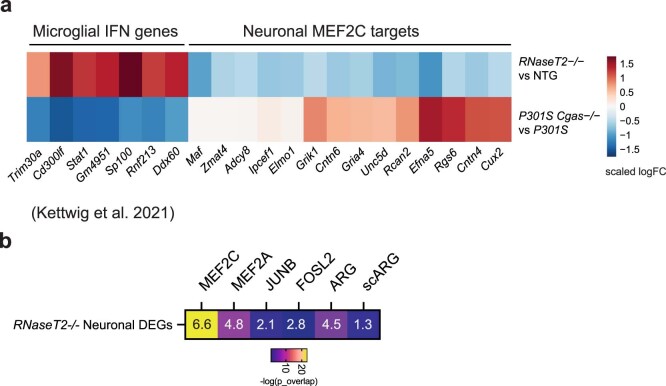
Downregulation of neuronal MEF2C transcriptional network in *RNaseT2*^*−/−*^ interferonopathy (Related to Fig. 5). **a**. Heatmap of scaled log fold-change of microglia interferon genes and neuronal MEF2C targets in *RNaseT2−/−* vs Ntg and *P301S Cgas−/−* vs *P301S*. **b**. Statistical enrichment analysis of overlap between RNaseT2*−/−* neuronal DEGs and MEF2C, MEF2A, JUNB, FOSL2, activity-regulated genes, and single-cell activity regulated genes (scARG). Numbers in the heatmap refers to odds ratios, color refers to -log(overlapping_p_val) via Fisher’s exact test.

To determine how the neuronal MEF2C transcription network is affected by IFN-I hyperactivation, we examined a published snRNA-seq dataset of *RNaseT2*^−/−^ mice, a model of infantile-onset RNaseT2-deficient leukoencephalopathy with severe IFN-I neuroinflammation and cognitive impairment^44^. Elevated microglial IFN and altered expression of neuronal MEF2C target genes in *RNaseT2*^−/−^ brains overlapped with those reversed by *Cgas* deletion in *P301S* transgenic mice (Extended Data Fig. 8a). MEF2C target genes were overrepresented in neuronal DEGs of RNaseT2 deficiency that was not observed for targets of MEF2A, JUNB, FOSL2 and activity-regulated genes (Extended Data Fig. 8b).

**Fig. 6:**
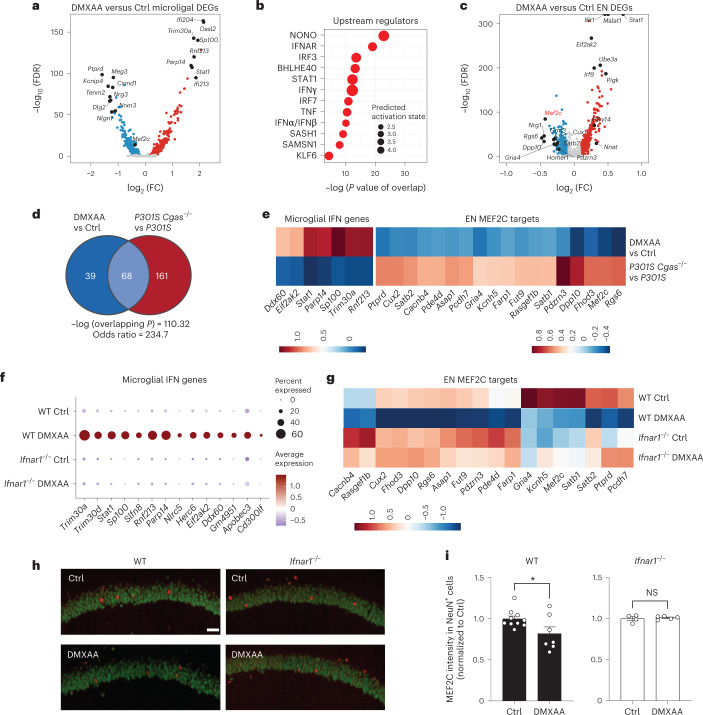
STING activation elevates microglial IFN-I and diminishes the neuronal MEF2C transcription network. **a**, Volcano plot showing representative DEGs in microglia of DMXAA-treated versus control WT mouse hippocampi; log_2_ FC of >0.1 or <−0.1 and FDR < 0.05. **b**, Ingenuity Pathway Upstream Regulator Analysis using DEGs from **a**. **c**, Volcano plot showing representative DEGs in ENs of DMXAA-treated versus control WT mouse hippocampi; log_2_ FC of >0.1 or <−0.1 and FDR < 0.05. **d**, Venn diagram showing the overlap of MEF2C target genes in DMXAA-treated versus control ENs and those in *P301S Cgas*^–/–^ versus *P301S* ENs in the hippocampus. **e**, Heat map of log_2_ FC of overlapping differentially expressed MEF2C target genes in DMXAA-treated versus control WT and in *P301S Cgas*^–/–^ versus *P301S* ENs in the hippocampus; scale, log_2_ FC. **f**, Dot plot showing microglial IFN gene expression in DMXAA-treated and control WT and *Ifnar1*^−/−^ mouse hippocampi. **g**, Downregulation of MEF2C target genes in WT neurons treated with DMXAA was rescued in *Ifnar1*^−/−^ neurons; scale, average gene expression. **h**, Representative ×25 confocal images of immunostaining of MEF2C and NeuN in the CA1 pyramidal layer of the mouse hippocampus in control and DMXAA-injected WT and *Ifnar1*^−/−^ mice; scale bar, 50 μm. **i**, Mean intensity of MEF2C in MEF2C^*+*^NeuN^+^ neurons in the CA1 pyramidal layer. Each circle represents the average intensity measurement of three images per animal. Data are reported as mean ± s.e.m. WT: *n* = 10 control and *n* = 7 DMXAA; *Ifnar1*^−/−^: *n* = 4 control and *n* = 5 DMAXX; *P = 0.0367. Data were analyzed by two-tailed unpaired *t*-test.

**Extended Data Fig. 9:**
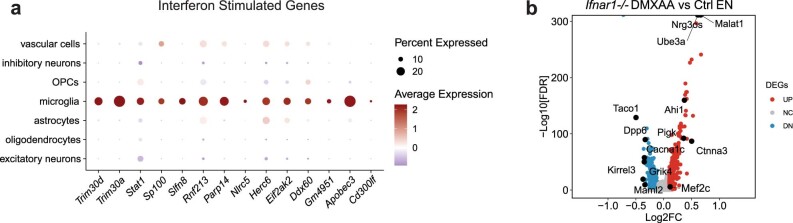
Microglial gene expression changes in response to DMXAA treatment in WT and *Ifnar1*^*−/−*^ mice (Related to Fig. 6). **a**. Dot plot showing expression of interferon-stimulated genes across cell types. **b**. Volcano plot showing representative DEGs in microglia of DMXAA treated vs control *Ifnar1*^*−/−*^ mouse hippocampi. (log2FC > 0.1, FDR < 0.05).

We next asked whether IFN-I activation is sufficient to suppress the neuronal MEF2C transcriptional network. To induce chronic IFN-I activation, we injected wild-type (WT) and *Ifnar1*^–/–^ mice with five treatments of 5,6-dimethylxanthenone-4-acetic acid (DMXAA), a STING agonist, over 12 d, followed by snRNA-seq of hippocampal tissues (Supplementary Fig. 2). In WT mice, microglia had the strongest IFN response to STING activation of all cell types (Extended Data Fig. 9a). Among the top DEGs in microglia were IFN-stimulated genes (*Ifi204*, *Oasl2*, *Trim30a*, *Sp100*, *Stat1* and *Rnf213*) with predicted activation of IRF3, IFNAR and STAT1 (Fig. 6a,b and Supplementary Table 9). In ENs, *Mef2c* and some of its target genes (*Rgs6*, *Dpp10*, *Gria4* and *Pdzrn3*) were among the top downregulated DEGs (Fig. 6c and Supplementary Table 9). The MEF2C target genes changed by DMXAA overlapped with those altered by *Cgas* deletion in tauopathy mice (Fig. 6d). *Cgas* deletion exerted opposite effects on microglial IFN-I expression and the MEF2C transcription network to those of STING activation (Fig. 6e).

The effects of IFN-I signaling on the MEF2C network were determined by comparing single-nuclei transcriptomes of WT and *Ifnar1*^−/−^ mice. Ablation of IFNα/IFNβ receptor 1 (IFNAR1) abolished the microglial IFN-I response and suppressed the neuronal MEF2C transcriptional network (Fig. 6f,g, Extended Data Fig. 9b and Supplementary Table 9). We quantified MEF2C expression in the CA1 region. DMXAA decreased MEF2C immunoreactivity in NeuN^+^ cells of WT hippocampi. No difference was observed in *Ifnar1*^−/−^ hippocampi (Fig. 6h,i). Thus, the neuronal MEF2C transcriptional network is suppressed by IFN-I activation.

### Inhibiting cGAS protects synapses and cognition in tauopathy

**Extended Data Fig. 10:**
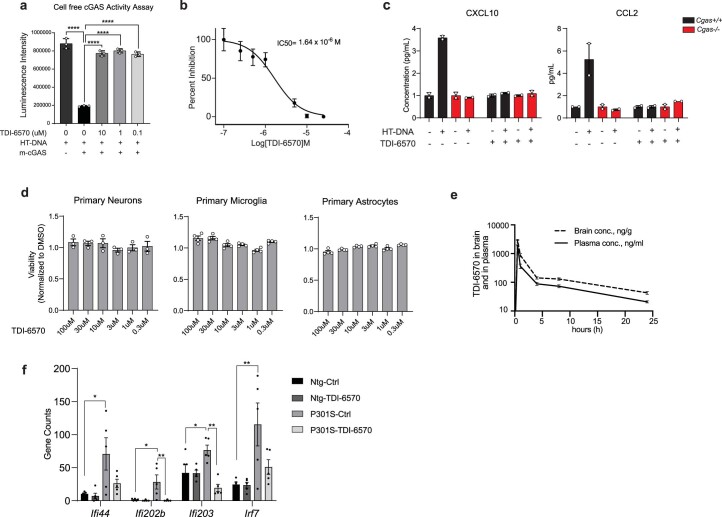
TDI-6570 is a non-toxic specific brain permeable cGAS inhibitor (Related to Fig. 7). **a**. Inhibitory effects of TDI-6570 (10–0.1 µM concentration) on mouse cGAS/HT-DNA-catalyzed conversion of ATP and GTP to produce cGAMP measured by remaining ATP concentrations in the reactions using ATP Glo assay. Data are reported as mean ± SD. n = 3; **** p < 0.0001. One-way ANOVA. **b**. Dose-dependent inhibition of cGAS activity in BV2 IfnB reporter line in response to HT-DNA. IC_50_ = 1.64 µM. Data are reported as mean ± SD. n = 4 per condition. **c**. Quantification of CXCL10 and CCL2 proteins by MagPix multiplex ELISA in culture medium supernatants of WT and *Cgas*^*−/−*^ primary mouse microglia treated with HT-DNA and TDI-6570 as indicated. n = 2 biologically independent samples. **d**. Cellular viability of mouse primary neurons (n = 3), microglia (n = 4) and astrocytes (n = 4) cultured in presence of TDI-6570 (0.3–100 µM concentration) for 24 h. Data are reported as mean ± SEM. **e**. Concentrations (in log10) of TDI-6570 in brain and plasma of C57BL/6 mice after a single dose (50 mg/kg) IP administration (n = 3) as determined by LC-MS/MS analysis. **f**. Bar plots of normalized counts show downregulation of interferon-inducible genes in the hippocampi of Ntg or *P301S* mice treated with 600 mg/kg TDI-6570 or Ctrl diets. Data are reported as mean ± SEM. n = 5 per condition. *Ifi44*: Ntg-Ctrl vs P301S-Ctrl * p = 0.0198; *Ifi202b*: Ntg-Ctrl vs P301S-Ctrl * p = 0.0125, P301S-Ctrl vs P301S-TDI-6570 ** p = 0.0091; *Ifi203*: Ntg-Ctrl vs P301S-Ctrl * p = 0.0480, P301S-Ctrl vs P301S-TDI-6570 ** p = 0.0011; *Irf7*: Ntg-Ctrl vs P301S-Ctrl * p = 0.0097. One-way ANOVA followed with Tukey’s multiple comparisons test for each gene.

We asked whether inhibiting cGAS ameliorates tauopathy-induced defects. *Cgas*^–/–^ mice were fertile and exhibited no deficits. Using in silico calculations, we predicted that TDI-6570, a specific cGAS inhibitor^25^, possesses high gastrointestinal absorption and brain permeability (Supplementary Table 10). We determined that TDI-6570 diminishes HT-DNA-induced cGAS activation and cGAS-dependent IFN responses in a dose-dependent manner with a half-maximal inhibitory concentration (IC_50_) of 1.64 × 10^−6^ M (Extended Data Fig. 10a,b). *Cgas*^−/−^ microglia were used to establish the specificity of TDI-6570 (Extended Data Fig. 10c). Concentrations of TDI-6570 up to 100 μM caused no changes in viability of primary neuron, microglia and astrocyte cultures (Extended Data Fig. 10d). TDI-6570 enters the brain with a brain-to-plasma ratio of 1.97 and a half-life of 12.4 h in the brain and 10.3 h in plasma (Extended Data Fig. 10e and Supplementary Table 10). TDI-6570 was formulated into chow diet and fed to 6- to 7-month-old non-transgenic and *P301S* transgenic mice, which reduced the expression of IFN-stimulated genes in the hippocampi of *P301S* transgenic mice (Extended Data Fig. 10f).

**Fig. 7:**
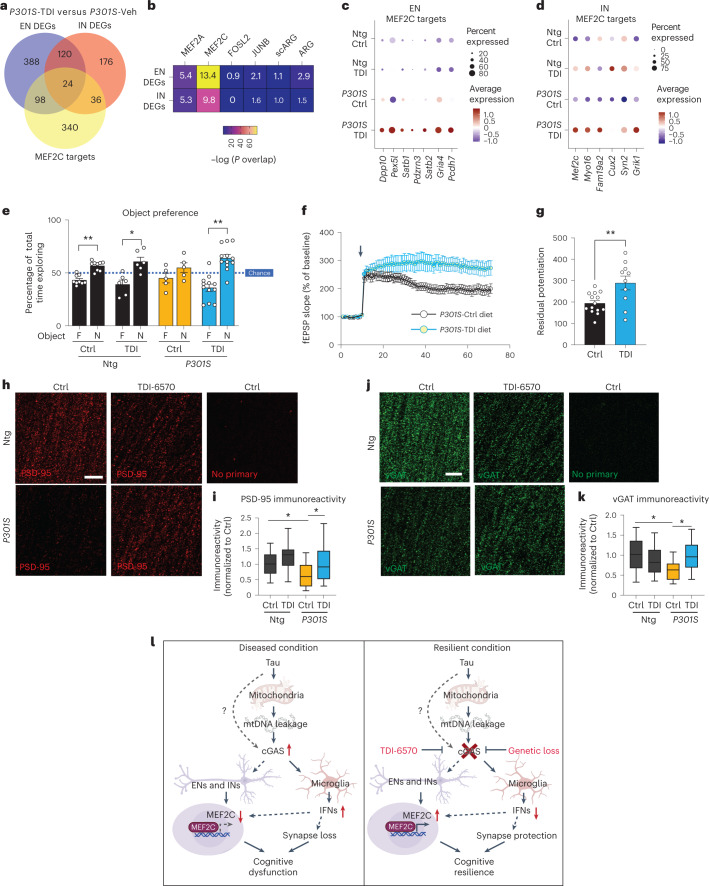
A brain-permeable cGAS inhibitor enhances the MEF2C network and protects against synapse loss and cognitive deficits in mice with tauopathy. **a**, Venn diagram of the overlaps in DEGs between *P301S* transgenic mice treated with TDI-6570 (*P301S*-TDI) and *P301S* transgenic mice treated with vehicle control (*P301S*-Veh) in ENs, INs and MEF2C target genes. **b**, Heat map showing the overlap between EN/IN DEGs and lists of TF target genes (MEF2A, MEF2C, FOSL2 and JUNB) and activity-induced DEGs (ARG and scARG). Numbers in each box represent the overlapping odds ratio. **c**, Dot plot showing the expression of significantly upregulated DEGs (FDR < 0.05, log_2_ FC ≥ 0.1) that are MEF2C targets in non-transgenic control (Ntg Ctrl), non-transgenic TDI-6570 (Ntg TDI), *P301S* transgenic control (*P301S* Ctrl) and *P301S* transgenic TDI-6570 (*P301S* TDI) EN clusters. **d**, Dot plot showing the expression of significantly upregulated DEGs (FDR < 0.05, log_2_ FC ≥ 0.1) that are MEF2C targets in non-transgenic control, non-transgenic TDI-6570, *P301S* transgenic control and *P301S* transgenic TDI-6570 IN clusters. **e**, Novel object recognition test for non-transgenic and *P301S* transgenic male mice fed 150 mg per kg (body weight) TDI-6570 or control diet for 3 months; F, familiar object; N, novel object. Data are reported as mean ± s.e.m.; *n* = 9 non-transgenic control, *n* = 6 non-transgenic TDI-6570, *n* = 5 *P301S* trasgenic control, *n* = 12 *P301S* transgenic TDI-6570. Non-transgenic control: **P = 0.00181; non-transgenic TDI-6570: *P = 0.0422; *P301S* transgenic TDI-6570: **P = 0.00167. Data were analyzed by two-tailed paired *t*-test for each condition. **f**, fEPSPs were recorded in the CA1 region, and a TBS protocol was applied (arrow) to the CA3 pathway to induce LTP. Data are reported as mean ± s.e.m. One to three slices per mouse were used; control, *n* = 13; TDI-6570, *n* = 9. **g**, LTP magnitude was calculated from the normalized mean fEPSP slope 50–60 min after TBS was applied. Data are reported as mean ± s.e.m.; one to three slices per mouse were used; *n* = 13 control; *n* = 9 TDI-6570; **P = 0.0058. Data were analyzed by two-tailed unpaired *t*-test. **h**, Representative confocal images of the hippocampal CA1 striatum radiatum labeled with PSD-95 antibody; scale bar, 10 μm. **i**, Mean intensity of PSD-95 puncta measured in the CA1 striatum radiatum. The center line is the median, box limits are the 25th to 75th percentiles, and whiskers are the minimum to maximum. Three to five images were taken per animal; *n* = 13 non-transgenic control, *n* = 12 non-transgenic, *n* = 9 TDI-6570, *n* = 12 *P301S* TDI-6570. Non-transgenic control versus *P301S* control: *P = 0.03, *P301S* control versus *P301S* TDI-6570: *P = 0.043. Data were analyzed by a two-way ANOVA mixed model. **j**, Representative confocal images of the hippocampal CA1 striatum radiatum labeled with vGAT antibody (scale bar = 10 μm). **k**, Mean intensity of vGAT puncta measured in the CA1 striatum radiatum. The center line is the median, box limits are the 25th to 75th percentiles, and whiskers are the minimum to maximum. Three to five images were taken per animal; *n* = 12 non-transgenic control, *n* = 11 non-transgenic TDI-6570, *n* = 8 *P301S* control, *n* = 13 *P301S* TDI-6570. Non-transgenic control versus *P301S* control: *P = 0.045, *P301S* control versus *P301S* TDI-6570: *P = 0.032. Data were analyzed by a two-way ANOVA mixed model. **l**, Working model illustrating the cGAS–IFN–MEF2C axis in tauopathy. In disease/vulnerable conditions, pathogenic tau activates the cGAS-dependent IFN response via mtDNA leakage in microglia and a reduction of the MEF2C transcriptional network in ENs and INs, resulting in cognitive dysfunction. Loss of cGAS reduces the IFN response in microglia and enhances the MEF2C transcriptional network, resulting in cognitive resilience.

To determine the efficacy of TDI-6570 against tau-mediated neurotoxicity, we fed a cohort of 6-month-old non-transgenic and *P301S* transgenic mice with 150 mg per kg (body weight) TDI-6570 or control diet for 3 months. The effects of TDI-6570 on ENs and INs were examined by snRNA-seq (Supplementary Fig. 3). *Mef2c* was a top upregulated gene in the IN clusters (Supplementary Table 11). Similar to *Cgas* deletion, we observed a strong overlap between DEGs and MEF2C target genes in ENs and INs (Fig. 7a,b). MEF2C target genes, but not the target genes of MEF2A, the neuronal activity-regulating TFs FOSL and JUNB nor activity-induced genes (ARG and scARG), were enriched in *P301S* TDI-6570 DEGs of ENs and INs (Fig. 7b). MEF2C target genes were among the top upregulated genes in *P301S* TDI-6570 versus *P301S* EN and IN clusters (Fig. 7c,d).

We performed novel object recognition to evaluate the impacts of cGAS inhibition on memory. *P301S* transgenic mice fed the control diet showed a defect in recognizing the novel object (Fig. 7e). This defect was rescued in mice fed TDI-6750. Given the protective effects of *Cgas* deletion on synapses in tauopathy, we asked if cGAS inhibition alters hippocampal synaptic plasticity and integrity of *P301S* transgenic mice. We performed TBS-induced LTP in the CA1 region of *P301S* transgenic mice fed control or TDI-6570 diets. Mice fed TDI-6570 had increased LTP magnitude (Fig. 7f,g). Immunofluorescence staining using antibodies to PSD-95 for excitatory synapses and vesicular GABA transporter (vGAT) for inhibitory synapses showed that chronic TDI-6570 treatment rescued *P301S*-dependent synapse loss (Fig. 7h–k). Thus, pharmacological inhibition of cGAS protected against synapse loss and cognitive deficits in the *P301S* tauopathy model, likely via enhancing the MEF2C transcriptional network and associated cognitive resilience.

## Discussion

Our study linked a hyperactive cGAS–IFN antiviral response with diminished MEF2C-associated cognitive resilience. In disease, cGAS hyperactivation promoted a microglial IFN-I response, reduced neuronal MEF2C transactivation and rendered loss of cognitive resilience. cGAS ablation diminished the microglial IFN-I response and enhanced the neuronal MEF2C transcription network and associated cognitive resilience against tau pathology (Fig. 7l). Inhibiting cGAS with TDI-6570 enhanced the expression of MEF2C target genes and restored synaptic integrity and memory, supporting the therapeutic potential of targeting the cGAS–MEF2C axis.

Activation of IFN responses in AD brains and mouse models is implicated in complement-associated synapse loss^8^. However, the immune activator remains elusive. Our analyses of two datasets revealed that tau induced a strong cGAS activation and IFN-I response in mice with tauopathy. Elevated levels of pTBK1 in AD brains further suggest that cGAS–STING activation underlies the IFN responses in AD. *Cgas* deletion in tau-stimulated microglia mitigated IFN and inflammatory signaling, supporting cGAS–STING–pTBK1 as an activator of the IFN-I response. Our snRNA-seq data showed that, rather than abolishing tau-induced microglial responses to tauopathy, *Cgas* deletion fine-tunes the microglial response-specific reduction of inteferon-related genes, with most DAM responses largely unaffected. The protective effects of cGAS inhibition support the feasibility of inhibiting the disease-enhancing microglial response.

*Cgas* is expressed in microglia, and our in vitro studies suggest that tau induces cGAS-dependent IFN signaling in microglia. As the sensor of cytosolic DNA, cGAS could be activated by leakage of DNA from the mitochondria or nucleus^22,23^. We showed that, after phagocytosis, tau is found in mitochondria and lysosomes. Tau entry into mitochondria may cause leakage of mtDNA into the cytosol to activate cGAS–STING. We found that tau fibrils trigger mtDNA release into the cytosol. By depleting mtDNA, we showed that tau-induced IFN responses were reduced in a dose-dependent manner in mtDNA-depleted microglia. TDP-43 overexpression also leads to release of mtDNA and cGAS–STING-induced neuroinflammation^13^. TDP-43 may enter the mitochondrial matrix via the mitochondrial import inner membrane translocase TIM22, which leads to destabilization of mitochondria and mtDNA leakage^13^. Whether TIM22 or other mitochondrial translocases are involved in the entry of tau into mitochondria is unknown. Mitochondrial stress induced by tau’s entry into mitochondria may directly or indirectly lead to leakage of mtDNA. Although our current evidence does not support the contribution of cytosolic DNA of nuclear origin, such as CCF, to cGAS–STING activation in cultured microglia, we cannot exclude its contribution in other cell types and in aging brains.

Protection by cGAS inactivation was associated with enhancement of the transcriptional network of a cognitive resilience gene, *Mef2c*. *MEF2C* is an AD risk gene, and variants within the *MEF2C* locus have been associated with differences in human intelligence^41,45,46^. In neurons, besides its role in neurodevelopment^37,47^, the MEF2 transcriptional network is upregulated in a subpopulation of ENs of the prefrontal cortex of resilient individuals in the presence of AD pathology and was the most predictive of end-stage cognition^16^. In our snRNA-seq analyses of DEGs in ENs and INs, *Mef2c* was one of the top hits upregulated by *Cgas* deletion. Both *Cgas* deletion and pharmacological inhibition induced the expression of MEF2C target genes in neurons, suggesting that upregulated *Mef2c* underlies the protective mechanism. Among the MEF2C target genes, *Cgas* deletion markedly altered a network of genes in hippocampal ENs and INs involved in axonal guidance, dendritic growth, synaptic maintenance, calcium signaling/homeostasis and neuronal excitability. These findings are consistent with the observation that MEF2C overexpression in *P301S* transgenic mice with tauopathy ameliorated hyperexcitability^16^, a hallmark of network dysfunction often observed in AD mouse models and a subset of individuals with AD^48^.

Emerging evidence supports the interplay of antiviral signaling and the MEF2C transcription network under various neurological conditions. Microglial MEF2C expression is decreased in brain aging and AD mouse models in an IFN-dependent manner^49,50^. MEF2C is downregulated in neurons of individuals with HIV-associated dementia^51^. In mice with RNaseT2 deficiency^44^, a form of type I interferonopathy^52^, we found that the neuronal MEF2C expression network is downregulated. In human AD, lower expression of neuronal MEF2C is associated with an elevated microglial IFN-I response. In our working model, under the disease condition, aberrant microglial cGAS–STING–IFN activation may drive neuronal MEF2C deficiency and compromise cognitive resilience (Fig. 7l). By contrast, inhibition of cGAS enhanced the MEF2C transcriptional network and rescued cognitive deficits despite similar tau pathology, enhancing cognitive resilience (Fig. 7l). While cGAS is mostly expressed in microglia, the neuronal STING–IFN axis directly regulates neuronal activity in peripheral sensory neurons^53^, supporting a potential role for the neuronal cGAS–STING–MEF2C axis. Using a STING agonist regimen, we showed that activation of the STING–IFN axis is sufficient to downregulate neuronal *Mef2c* and its targets in an IFNAR1-dependent manner. Because IFNAR1 is expressed in neurons and microglia, cell-type-specific deletion of *Cgas* and/or *Ifnar1* is needed to dissect the link between cGAS and regulators of cognitive resilience and neuronal excitability.

In AD brains, accumulation of plaques and tangles precedes clinical symptoms by decades, indicating a long period of cognitive resilience in healthy aging. Harnessing the brain’s intrinsic cognitive resilience mechanisms could lead to treatments. Our finding that cGAS inactivation protects against tau toxicity supports an exciting new class of strategies after the onset of plaque and tangle pathologies. Activation of STING signaling is linked to other neurodegenerative diseases, and inhibiting STING activation protects against deleterious effects of IFN responses in Parkinson’s disease, Huntington’s disease and amyotrophic lateral sclerosis^11–13^. This, along with the fact that *Cgas*^−/−^ mice are healthy and fertile, supports the inhibition of cGAS as a promising AD therapeutic strategy.

## Methods

### Mice

Mice were housed no more than five per cage, given ad libitum access to food and water and housed in a pathogen-free barrier facility at 21–23 °C with 30–70% humidity on a 12-h light/12-h dark cycle. *P301S* transgenic mice (The Jackson Laboratory, 008169) were crossed with *Cgas*^−/−^ mice (The Jackson Laboratory, 026554) to generate *P301S Cgas*^+/−^ mice, and subsequent crossing of F1 litters generated *Cgas*^+/+^, *Cgas*^+/−^ and *Cgas*^−/−^ mice and their corresponding *P301S* transgenic littermates. Mice of both sexes were used for behavioral, histological and biochemical analyses. Mice underwent behavioral testing at 7–8 months of age and had not been used for any other experiments. At 9–10 months of age, the same mice were used for pathology and RNA-seq studies. For TDI-6570 in vivo treatment, *P301S* and non-transgenic littermate mice at 6–7 months of age were used for diet experiments and were assayed for behavior and histology at 9–10 months. For the DMXAA treatment experiment, WT C57BL/6J (000664) and *Ifnar1*^−/−^ (028288) mice of 3 months of age were purchased from The Jackson Laboratory. All mouse protocols were approved by the Institutional Animal Care and Use Committee, University of California, San Francisco, and Weill Cornell Medicine.

### Bulk RNA-seq

Brains recovered from freshly perfused mice were dissected to isolate hippocampi and cortices. Hippocampi were split in two and frozen at −80 °C until experimentation. To isolate RNA, hippocampi were thawed on ice for 30 min and homogenized. Briefly, hippocampi were passed through a 21-gauge needle in a solution of RLT buffer containing 1% β-mercaptoethanol. After homogenization, samples were centrifuged briefly and frozen at −80 °C overnight. The next day, samples were thawed on ice and centrifuged at 4 °C for 6 min at 20,000*g*. RNA isolation was performed on hippocampal lysates according to the manufacturer’s protocol (RNeasy mini-kit, Qiagen). Isolated RNA was submitted to the Weill Cornell Medicine Genomics Core for analysis of RNA quality and integrity. All samples passed quality control, and RNA-seq libraries were prepared for sequencing using NovaSeq.

### Nuclei isolation from frozen mouse hippocampi

Nuclei isolation from frozen mouse hippocampi was adapted from a previous study, with modifications^54,55^. All procedures were performed on ice or at 4 °C. In brief, postmortem brain tissue was placed in 1,500 µl of Sigma nuclei PURE lysis buffer (Sigma, NUC201-1KT) and homogenized with a Dounce tissue grinder (Sigma, D8938-1SET) with 20 strokes with pestle A and 15 strokes with pestle B. The homogenized tissue was filtered through a 35-µm cell strainer, centrifuged at 600*g* for 5 min at 4 °C and washed three times with 1 ml of PBS containing 1% bovine serum albumin (BSA), 20 mM DTT and 0.2 U µl^−1^ recombinant RNase inhibitor. Nuclei were then centrifuged at 600*g* for 5 min at 4 °C and resuspended in 800 µl of PBS containing 0.04% BSA and 1× DAPI, followed by fluorescence-activated cell sorting to remove cell debris. The sorted suspension of DAPI-stained nuclei was counted and diluted to a concentration of 1,000 nuclei per µl in PBS containing 0.04% BSA.

### Droplet-based snRNA-seq

For droplet-based snRNA-seq, libraries were prepared with Chromium Single Cell 3′ Reagent kits (v3; 10x Genomics, PN-1000075), according to the manufacturer’s protocol. The snRNA-seq libraries were sequenced on a NovaSeq 6000 sequencer (Illumina) with 100 cycles.

### Analysis of droplet-based snRNA-seq data from brain tissue

Gene counts were obtained by aligning reads to the mm10 genome with Cell Ranger software (v.3.1.0; 10x Genomics). To account for unspliced nuclear transcripts, reads mapping to pre-mRNA were counted. Cell Ranger 3.1.0 default parameters were used to call cell barcodes. We further removed genes expressed in no more than two cells, cells with unique gene counts over 4,000 or less than 200 and cells with a high fraction of mitochondrial reads (>5%). Potential doublet cells were predicted using DoubletFinder^26^ for each sample separately, with high-confidence doublets removed. Normalization and clustering were done with the Seurat package v3.2.2 (ref. ^56^). In brief, counts for all nuclei were scaled by the total library size multiplied by a scale factor (10,000) and transformed to log space. A set of 2,000 highly variable genes was identified with SCTransform from the sctransform R package in the variable stabilization mode. This returned a corrected unique molecular identifier count matrix, a log-transformed data matrix and Pearson residuals from the regularized negative binomial regression model. Principal-component analysis was done on all genes, and t-distributed stochastic neighbor embedding was run on the top 20 principal components. Cell clusters were identified with the Seurat functions FindNeighbors (using the top 20 principal components) and FindClusters (resolution = 0.02). In this analysis, the neighborhood size parameter pK was estimated using the mean variance-normalized bimodality coefficient (BCmvn) approach, with 20 principal components used and pN set as 0.25 by default. For each cluster, we assigned a cell-type label using statistical enrichment for sets of marker genes^57,58^ and manual evaluation of gene expression for small sets of known marker genes. Differential gene expression analysis was done using the FindMarkers function and MAST^59^. To identify gene ontology and pathways enriched in the DEGs, DEGS were analyzed using the MSigDB gene annotation database^60,61^. To control for multiple testing, we used the Benjamini–Hochberg approach to constrain the FDR. For trajectory analysis, Seurat objects were converted to cds objects and analyzed using Monocle 3 (refs. ^29,62,63^). Moran’s I spatial autocorrelation analysis was performed to identify gene modules significantly associated with microglial trajectory. Enrichment analysis of target genes was performed with the GeneOverlap package in R. Briefly, the MEF2C, MEF2A, JUNB and FOSL2 target gene lists and human cognitive resilience gene lists (kindly shared by L. -H. Tsai, Massachusetts Institute of Technology) and *P301S Cgas*^−/−^ versus *P301S* EN/IN DEG lists were used as the input for the comparison. The results include the overlapping *P* value and the odds ratio that examines the association between the two datasets. In our analyses of snRNA-seq data, we excluded the data from potential doublet cells as identified by DoubletFinder. For the *Cgas* genetic cohort snRNA-seq, we sequenced and integrated samples from *Cgas*^+/+^ (*n* = 5), *Cgas*^+/−^ (*n* = 2), *Cgas*^−/−^ (*n* = 2), *P301S Cgas*^+/+^ (*n* = 6), *P301S Cgas*^+/–^ (*n* = 6) and *P301S Cgas*^–/–^ (*n* = 6) mice. *Cgas*^+/−^ and *Cgas*^−/−^ genotypes had sample sizes of two and thus were not included in downstream analyses. For the analysis of snRNA-seq data for TDI-6570 diet- versus control diet-fed mice, one mouse (P301S_Ctrl_3) was identified as an outlier and excluded.

### Western blots

For mouse brain samples, half hippocampi were mechanically homogenized on ice in RIPA buffer containing protease and phosphatase inhibitors (Millipore Sigma). Fifty micrograms of hippocampal lysates was loaded onto NuPage Bis-Tris gels (Thermo Fisher) and run in SDS running buffer at 150 V for ~2.5 h. Gels were transferred to nitrocellulose membranes (Bio-Rad) overnight in a cold room. Membranes were washed three times for 10 min each in TBS with 0.01% Triton X-100 (TBST) and blocked for 1 h in 5% milk in TBST. Appropriate primary antibodies were diluted in 1% milk in TBST and incubated at 4 °C overnight. The following day, membranes were washed three times for 10 min each in TBST and incubated with appropriate secondary antibodies in 1% milk in TBST for 1 h at room temperature. Membranes were washed again to minimize nonspecific binding, treated with ECL (Bio-Rad) for 60 s and developed in a dark room or using a Bio-Rad imager. Blots were scanned at 300 d.p.i. and quantified using ImageJ.

For human brain samples, frontal cortex lysates were prepared as previously described^64^. Briefly, human brain tissues were lysed in RIPA buffer containing protease inhibitor cocktail (Sigma), 1 mM phenylmethylsulfonyl fluoride (Sigma), phosphatase inhibitor cocktail (Roche) and histone deacetylase inhibitors, including 5 mM nicotinamide (Sigma) and 1 mM trichostatin A (Sigma). After sonication, lysates were centrifuged at 17,000*g* at 4 °C for 15 min. Supernatants were collected, and protein concentration was measured by bicinchoninic acid (BCA) assay (Thermo Scientific).

For cultured primary microglia, 1 million to 2 million cells were lysed in M-PER mammalian protein extraction reagent (Thermo Scientific) supplemented with HALT protease and phosphatase inhibitor cocktail (Thermo Scientific) and 150 mM NaCl. Samples were rotated at 4 °C for 10 min. Lysates were cleared by centrifugation at 16,000*g* for 15 min at 4 °C. Protein concentration was measured by BCA assay.

### Antibodies

Antibodies used in immunofluorescence analyses were as follows. Secondary antibodies used were Alexa Fluor donkey anti-rabbit/goat 488 and anti-mouse 555 (Invitrogen) and donkey anti-goat 555 at a dilution of 1:500 (Jackson ImmunoResearch).

Primary antibodies included anti-STING (clone D2P2F, Cell Signaling Technology, 13647; 1:300), anti-IBA1 (Abcam, ab5076; 1:500), anti-PSD-95 (clone 6G6-1C9, Millipore, MAB1596; 1:500), anti-vGAT (Millipore, Ab5062; 1:500), anti-pSTAT1 (Clone 58D6, Cell Signaling Technology, mAb9167; 1:500), anti-NRG1 (clone 7D5, Invitrogen, MA5-12896; 1:100), MC1 (a generous gift from P. Davis (Feinstein Institute for Medical Research); 1:400), anti-MEF2C (clone 681824, R&D Systems, MAB6786; 1:200), anti-NeuN (Millipore, ABN78; 1:500) and anti-γ-H2.AX (Abcam, ab2893; 1:500).

Primary antibodies used for western blotting were TBK1 (clone D1B4, Cell Signaling Technology, 3504; 1:1,000), pTBK1 (clone D52C2, Cell Signaling Technology, 5483; 1:500), GAPDH (clone 6C5, Millipore, MAB374 (1:10,000) and GeneTex, GTX100118 (1:10,000)), TOMM20 (clone 4F3, Millipore Sigma, ST1705; 1:1,000) and lamin A/lamin B1/lamin C (clone EPR4068, Abcam, ab108922; 1:1,000).

Secondaries used for western blotting were anti-rabbit horseradish peroxidase (Calbiochem, 401393; 1:2,000) or anti-mouse horseradish peroxidase (Calbiochem, 401253; 1:2,000).

For immunogold labeling electron microscopy, an antibody to tau (Agilent Technologies, A0024; 1:1,000) was used.

### Immunofluorescence

Hemibrains from transcardially perfused mice were placed in 4% paraformaldehyde for 48 h, followed by 30% sucrose in PBS for 48 h at 4 °C. Sections were cut coronally at 40 µm using a freezing microtome (Leica) to produce eight to ten free-floating series per mouse and placed in cryoprotective medium at –20 °C until use. All washing steps were 5 min long and were performed three times. Sections were washed in TBST (0.01% Triton X-100), permeabilized with TBST (0.5% Triton X-100) for 15 min and washed again. Sections were then placed in antigen-unmasking solution (citrate buffer, pH 6.0; h-3300) and placed in a 90 °C incubator for 30 min, when applicable. Sections were washed and then blocked in 10% normal donkey serum (Vector, BMK-2202) in TBST for 2 h at room temperature. Primary antibodies were diluted in 5% normal donkey serum in TBST and incubated overnight at 4 °C. The following day, sections were washed thoroughly and incubated in appropriate secondary antibodies (1:500; Invitrogen) for 1 h. Sections were washed, mounted on slides and imaged. For pSTAT1 imaging, the CA1 region of mouse brain sections was imaged with a Zeiss Apotome ×20 objective (Carl Zeiss). Images were taken with *z* stacks of 7-µm intervals at a 1-µm step size. For MC1 imaging, images were acquired with a Keyence BZ-X700 microscope using a ×10 objective. Quantification was done with ImageJ software (NIH) using percent area for MC1. For MEF2C, images of the mouse brain CA1 pyramidal region were acquired using a ×25 objective on a Zeiss LSM 880 confocal microscope (Carl Zeiss). A 4 × 1 tile scan and *z* stack of 12 µm at a step size of 3 µm was used to generate a maximum intensity projection stitched image for each section. For PSD-95, vGAT and NRG1, slides were imaged using a Zeiss LSM 880 confocal microscope. Quantification was done using ImageJ software (NIH) using percent area based on thresholding determined using negative and positive controls. For higher resolution, images were acquired with an LSM 880 confocal microscope and Zen Black image acquisition software with a ×40 objective. The CA1 region of the hippocampus was imaged with a 1-µm interval *z* stack over a total distance of 15 µm per slice and a 2 × 2 tile scan. Final images were processed with maximum intensity projection.

### Behavioral tests

In all behavioral tests, *Cgas*^+/+^, *Cgas*^+/−^ and *Cgas*^−/−^ mice were compared to their respective *P301S* transgenic littermates. Male and female mice were tested on separate days, and experimenters were blinded to mouse genotypes throughout the experiments. For experiments involving TDI-6570 treatment, male *P301S* and non-transgenic littermates fed with TDI-6570 or the control diet were used.

#### Morris water maze

The water maze consisted of a pool (122 cm in diameter) containing opaque water (20 ± 1 °C) and a platform (10 cm in diameter) 1.5 cm below the surface. Four different images were posted on the walls of the room as spatial cues. Hidden platform training (days 1–6) consisted of 14 sessions (2 sessions per day 2 h apart), each with two trials. The mouse was placed into the pool at alternating quadrants for each trial. A trial ended when the mouse located the platform or after 60 s had elapsed. Mice received 6 d of hidden platform training before the 24-h and 72-h probe trials. For probe trials, the hidden platform was removed, and mice were allowed to swim for 60 s. Visible platform testing was done 24 h after the last probe trial. Performance was measured with an EthoVision video tracking system (Noldus Information Technology).

#### Elevated plus maze

The maze consisted of two 38 × 5 cm open arms without walls and two closed arms with walls 16.5 cm tall and 77.5 cm above the ground. Mice were moved to the testing room 1 h before testing to acclimate to the dim lighting. Mice were individually placed in the maze at the intersection of the open and closed arms and allowed to explore the maze for 10 min.

#### Open field

Mice were individually placed into automated activity chambers equipped with rows of infrared photocells connected to a computer (San Diego Instruments). Open field activity was recorded for 15 min. Recorded beam breaks were used to calculate total activity.

#### Novel object recognition test

Mice were habituated to opaque open field arenas (40 × 40 cm) for two 10-min trials spaced on the 2 d leading up to object recognition. At 24 h after the second arena habituation trial, two identical objects (glass jars) were placed in the center of each arena. Mice were allowed to explore these objects for a single 15-min trial. The subsequent day after object habituation, one of the identical objects was replaced with a novel object (DUPLO block structure) for a 15-min test period. Video recording and tracking (Ethovision v15, Noldus) was used to determine total distance moved. An experimenter blind to the groups manually scored the time mice spent exploring each object. Preference was calculated based on the total time an individual mouse spent exploring both objects.

### Electrophysiology

The brain was quickly dissected from anesthetized mice and placed into ice-cold dissection solution containing 210 mM sucrose, 2.5 mM KCl, 1.25 mM NaH_2_PO_4_, 25 mM NaHCO_3_, 7 mM glucose, 2 mM MgSO_4_ and 0.5 mM CaCl_2_ (gassed with 95% O_2_ and 5% CO_2_, pH ∼7.4). Horizontal slices (400-µm thickness) were made on a vibratome, and the slices were incubated for 30 min in artificial cerebral spinal fluid (ACSF) warmed to 35 °C containing 119 mM NaCl, 2.5 mM KCl, 26.2 mM NaHCO_3_, 1 mM NaH_2_PO_4_, 11 mM glucose, 1.3 mM MgSO_4_ and 2.5 mM CaCl_2_ (gassed with 95% O_2_ and 5% CO_2_, pH ∼7.4). Slices were then kept at room temperature in oxygenated ACSF until recordings were performed. Field recordings were performed in the dentate gyrus molecular layer of the acute horizontal brain slices placed in a recording chamber. Slices were submerged in oxygenated ACSF that was continuously perfused at 30 °C. The glass recording electrode (~3 MΩ pipette resistance) was filled with ACSF and lowered ~50 µm into the molecular layer of the dorsal blade of the dentate gyrus. A bipolar tungsten electrode (FHC), located ~150 µm from the recording electrode, was used to stimulate the perforant pathway inputs to the dentate gyrus. Stimulus pulses were generated by a Model 2100 Isolated Pulse Stimulator (A-M Systems). Responses were evoked every 30 s with stimulus intensities ranging from 5 to 40 µA with a 0.5-ms stimulus duration. After recording fEPSPs in response to 5- to 40-µA stimulation, the stimulus intensity was adjusted to evoke 30% of the maximal fEPSP slope to set the baseline for LTP recordings. LTP recordings were performed in the presence of picrotoxin (100 µM; Sigma). After recording baseline fEPSPs for 20 min, TBS was applied, which included 10 theta bursts applied every 15 s, and each theta burst consisted of 10 bursts (four pulses, 100 Hz) every 200 ms. The stimulus intensity was raised to a level that was 60% of the maximal fEPSP slope only during TBS and then returned to the stimulus intensity used during the baseline recording following TBS. The fEPSP slope was normalized to the baseline responses before LTP induction. Recordings were performed using a Multiclamp 700B amplifier (Molecular Devices), digitized at 10 kHz and acquired with WinLTP software (version 1.11b, University of Bristol) and analyzed using WinLTP software. Recordings and analyses were done blind to mouse genotype.

### Microglia culture and isolation

#### BV2 microglia culture

The BV2 microglia cell line was maintained in growth medium (DMEM; Thermo Fisher) supplemented with 10% one-shot fetal bovine serum (FBS; Gibco) and 1% penicillin–streptomycin (Life Technologies) in HERAcell 150i incubators (Caisson Labs) at 37 °C with 5% CO_2_. BV2 microglia were serially passaged once plates reached 80–90% confluency.

#### THP1 cell culture

THP1-Dual cells (thpd-nfis) and THP1-Dual KO-cGAS cells (thpd-kocgas) were purchased from InvivoGen. The THP1 cell lines were maintained in suspension in RPMI growth medium (Thermo Fisher) supplemented with 10% one-shot FBS (Gibco) and 1% penicillin–streptomycin (Life Technologies) in HERAcell 150i incubators (Caisson Labs) at 37 °C with 5% CO_2_. BV2 microglia were passaged every 3–4 d.

#### Isolation and culture of postnatal primary microglia

Primary microglial cells were collected from mouse pups at postnatal days 1–3. Briefly, the brain cortices were isolated and minced. Tissues were dissociated in 0.25% Trypsin-EDTA for 10 min at 37 °C and agitated every 5 min. Two hundred microliters of DNAse I (Millipore) was then added. Trypsin was neutralized with complete medium (DMEM; Thermo Fisher) supplemented with 10% heat-inactivated FBS (Hyclone), and tissues were filtered through 70-μm cell strainers (BD Falcon) and pelleted by centrifugation at 250*g*. Mixed glial cultures were maintained in growth medium at 37 °C and 5% CO_2_ for 7–10 d in vitro. Once bright, round cells began to appear in the mixed glial cultures. Recombinant mouse granulocyte–macrophage colony-stimulating factor (1 ng ml^–1^; Life Technologies) was added to promote microglia proliferation. Primary microglial cells were collected by mechanical agitation after 48–72 h and plated on poly-d-lysine-coated 24-well plates (Corning) in growth medium. Microglia were maintained in DMEM supplemented with 10% FBS, 100 U ml^–1^ penicillin and 100 μg ml^–1^ streptomycin. Immune stimulatory assays were performed 24 h after microglia plating with HT-DNA (Sigma; 1 μg ml^–1^) or tau fibrils (0N4R; 2 μg ml^–1^). RNA was isolated 6 h after stimulation (Zymo Research) and submitted to Novogene for analysis of RNA quality and integrity. All samples passed quality control, and RNA-seq libraries were prepared for sequencing using a HiSeq.

#### Human iPSC differentiation into microglia and tau stimulation

iPSCs used in this experiment were from an adult female, without any known diseases, and were purchased from WiCell (UCSD072i-1-3). iPSCs were passage 17 at the start of differentiation and were differentiated into macrophage progenitors via a 10-d protocol, as previously described^65^. Cells were then further differentiated into microglia-like cells via a 13-d culture with macrophage colony-stimulating factor (10 ng ml^–1^) and interleukin-34 (IL-34; 100 ng ml^–1^) in RPMI supplemented with 10% FBS^65^. Microglial identity was confirmed with positive staining for IBA1 (Abcam, ab5076) and TMEM119 (Sigma, HPA051870). iPSC-derived microglia were pretreated with 20 µM TDI-8246, 2 or 5 µM H-151 or 0.2% DMSO control for 6 h, followed by 0N4R tau fibril treatment (1 µg ml^–1^) overnight for 18–20 h.

### Generation of the IFNβ reporter line

BV2 microglia were transfected with mouse IFNβ reporter plasmid, pNiFty3-I-Lucia (Invivogen), using Lipofectamine 2000 (Thermo Fisher). Transfection medium was removed 6 h after transfection and replaced with complete growth medium. Selection with zeocin was conducted for 3 weeks, after which, resistant cells were plated as single clones in a 96-well plate (Corning). Genomic DNA was isolated from clones to confirm integration of luciferase reporter. Clones were then validated for the induction of IFN in response to cGAS agonists.

### Luciferase reporter assays

BV2 IFNβ reporter microglia were stimulated with 0–10 µg of HT-DNA (Sigma) or cGAMP (InvivoGen) or 2 µg ml^–1^ 0N4R tau fibrils. THP1-Dual and THP1-Dual KO-cGAS cells were stimulated with 0–10 µg of HT-DNA (Sigma). HT-DNA was delivered to cells using Lipofectamine 2000 (Thermo Fisher). Luciferase levels secreted in medium were measured using QUANTI-Luc (InvivoGen). Luminescence was measured on a BioTek Synergy hybrid reader.

### Secreted embryonic alkaline phosphatase reporter assays

THP1-Dual and THP1-Dual KO-cGAS cells were stimulated with 0–10 µg of HT-DNA (Sigma). HT-DNA was delivered to cells using Lipofectamine 2000 (Thermo Fisher). To measure NF-κB activation, secreted embryonic alkaline phosphatase levels in the cell medium were measured using QUANTI-Blue (InvivoGen). Absorbance was measured on a BioTek Synergy hybrid reader.

### Cell viability assays

Cell viability of BV2 microglia was measured 24 h after treatment using a CellTiter-Glo assay (Promega). Briefly, cells in 96-well plates were equilibrated at room temperature for 30 min and lysed with luciferase buffer. Luminescence was measured on a BioTek Synergy hybrid reader.

### Cell-free cGAS activity assay

Activities of compounds TDI-6570 (against mouse cGAS; 30 nM) and TDI-8246 (against human cGAS; 100 nM) were determined by measuring the conversion of ATP and GTP (100 µM each) to cGAMP in the presence of dsDNA (5 µg ml^–1^) in a reaction buffer composed of Tris-HCl (20 mM, pH 7.4), NaCl (150 mM), MnCl_2_ (0.2 mM, human cGAS) or MgCl_2_ (5 mM, mouse cGAS) and Tween 20 (0.01%) and the remaining ATP concentration using a Kinase-GloMax luminescent kinase assay (Promega), as previously described^25^. Briefly, 10 μl of a master mix of 0.4 mM ATP, 0.4 mM GTP and 0.02 mg ml^–1^ dsDNA in the reaction buffer supplemented with 2 mM DTT was added to reaction wells containing TDI-6570 or TDI-8246 (two times the desired concentration) in the same buffer (20 µl). Next, 10 μl of a 4× mouse cGAS (0.120 μM) or human cGAS (0.4 μM) solution in the reaction buffer supplemented with 2 mM DTT was added to appropriate wells. Similar sets of reactions without cGAS or the inhibitor were set by adding the buffer alone. The reactions with the plates sealed were incubated at 37 °C (1 h for mouse cGAS and 3 h for human cGAS) and stopped by the addition of 40 μl of Kinase-GloMax. Luminescence was recorded in relative light units (RLUs) using a Biotek Synergy H1 hybrid plate reader (BioTek). ATP depletion was normalized against the positive control (no cGAS) and negative control (with cGAS) as follows: percent inhibition = 100 × (RLU sample − RLU average negative control) ÷ (RLU average positive control – RLU average negative control).

### mtDNA leakage assay

BV2 cells (2 × 10^6^ to 3 × 10^6^) untreated or treated with 2 μg ml^–1^ 0N4R for 6 h were scraped and washed with PBS. Each sample was divided into two equal aliquots, and one aliquot was used for total DNA purification using a DNeasy Blood & Tissue kit (Qiagen). The other aliquot was subjected to digitonin-based cytosolic fractionation. Briefly, cell pellets were resuspended in 50 μl of digitonin lysis buffer (10 mM KCl, 5 mM MgCl_2_, 1 mM EDTA, 1 mM EGTA, 250 mM sucrose, 20 mM HEPES (pH 7.2) and 0.025% (wt/vol) digitonin) and incubated for 10 min on ice followed by a 15-min centrifugation at 15,000*g* at 4 °C. The cytosolic fraction was collected as supernatant and subjected to DNA purification using a QIAamp DNA micro kit (Qiagen). RT–qPCR was performed on both whole-cell extracts and cytosolic fractions using nuclear DNA primers (*Tert*) and mtDNA primers (*Dloop1* and *Nd2*), and the cycling threshold (*C*_*t*_) values obtained for mtDNA abundance for whole-cell extracts served as normalization controls for the mtDNA values obtained from the cytosolic fractions. The following primer pairs were used:

*Dloop1* forward: AATCTACCATCCTCCGTGAAACC

*Dloop1* reverse: TCAGTTTAGCTACCCCCAAGTTTAA

*Nd2* forward: CCATCAACTCAATCTCACTTCTATG

*Nd2* reverse: GAATCCTGTTAGTGGTGGAAGG

*Tert* forward: CTAGCTCATGTGTCAAGACCCTCTT

*Tert* reverse: GCCAGCACGTTTCTCTCGTT

### mtDNA depletion assay

BV2 IFNβ reporter microglia were treated with 50–100 ng ml^–1^ EtBr (Sigma-Aldrich) or 40–80 μg ml^–1^ ddC (Sigma-Aldrich) in DMEM supplemented with 10% FBS (Gibco), 100 U ml^–1^ penicillin and 100 μg ml^–1^ streptomycin for 7 d. On day 7, cells were detached from the plate by scraping. A portion of the cells was saved for mtDNA RT–qPCR assays. The rest of the cells were plated in DMEM-F12 supplemented with 100 U ml^–1^ penicillin and 100 μg ml^–1^ streptomycin and allowed to rest for 48 h. Cells were then treated with 0N4R tau fibrils or ABT-737 + QVD (10 μM each; SelleckChem) for 24 h before being assayed for luciferase reporter activity and cell viability. For mtDNA depletion RT–qPCR assays, DNA was extracted from cell pellets using a DNeasy Blood & Tissue kit (Qiagen). The ratio of mtDNA (*Nd2*) to genomic DNA (*Tert*) was measured by SybrGreen RT–qPCR (Bio-Rad).

### ELISA and multiplex bead-based immunoassay

Cell culture medium from cultured primary mouse microglia was collected 24 h after stimulation and cleared by centrifugation at 500*g* for 5 min. Supernatants were diluted 1:10 and assayed using a VeriKine-HS mouse IFNβ serum ELISA kit (PBL Assay Science) according to the manufacturer’s instructions. CXCL10 and CCL5 were measured with a MILLIPLEX MAP mouse cytokine/chemokine magnetic bead kit (Millipore) using a MAGPIX system. For heat map generation, the mean and standard deviation of expression over all samples were calculated for each cytokine, and the expression values were linearly transformed using the formula (concentration – mean) ÷ standard deviation.

### Electron microscopy

Electron microscopy experiments were performed by the Electron Microscopy Core Facility at Weill Cornell Medicine. Cells were washed with serum-free medium or appropriate buffer and fixed with a modified Karmovsky’s fixative consisting of 2.5% glutaraldehyde, 4% parafomaldehye and 0.02% picric acid in 0.1 M sodium cacodylate buffer (pH 7.2). After a secondary fixation in 1% osmium tetroxide and 1.5% potassium ferricyanide, samples were dehydrated through a graded ethanol series and embedded in situ in LX-112 resin (Ladd Research Industries). En face ultrathin sections were cut using a Diatome diamond knife (Diatome) on a Leica Ultracut S ultramicrotome (Leica). Sections were collected on copper grids and further contrasted with lead citrate. For immunolabeling, the sections were collected on 200-mesh nickel grids. Briefly, sections were rehydrated in PBS. Unreacted aldehydes were quenched with 50 mM glycine in PBS, followed by blocking for the host of secondary antibody (Aurion, EMS) for 15 min at room temperature. Primary antibody incubation was performed overnight at 4 °C in PBS + 0.2% BSA-c (PBS-c; Aurion, EMS). The next day, sections were washed six times in PBS-c and incubated with secondary antibody (Aurion gold conjugate 1:100 in blocking buffer) for 1–2 h at room temperature. Washes were performed with PBS-c and then water. Sections were then fixed in 2.5% glutaraldehyde in 0.1 M sodium cacodylate buffer, washed with PBS, contrasted with uranyl acetate and allowed to air dry after a final wash with water. Samples were viewed on a JEM 1400 electron microscope (JEOL) operated at 120 kV. Digital images were captured on a Veleta 2 K × 2 K CCD camera (Olympus-SIS).

### DMXAA treatment

DMXAA solution was freshly prepared before each injection by mixing DMXAA (purchased from Tocris Bioscience) in DMSO with PEG-400 and (2-hydroxypropyl)-β-cyclodextrin (5:60:35). WT and *Ifnar1*^−/−^ mice received intraperitoneal injections of DMXAA (WT, *n* = 10; *Ifnar1*^−/−^, *n* = 5) or vehicle (WT, *n* = 10; *Ifnar1*^−/−^, *n* = 5) every 3 d for a total of five injections over 12 d. For each treatment, the mice were weighed and injected 10 µl per g of drug or vehicle. The dose of DMXAA was 12.5 mg per kg (body weight) for the first four treatments and 6.25 mg per kg (body weight) for the last treatment. At 24 h after the last treatment, mice were perfused with PBS. The right hemispheres were dissected, and hippocampi were used for snRNA-seq. The left hemispheres were postfixed in 4% paraformaldehyde for immunohistochemistry.

### Synthesis of cGAS inhibitors

TDI-6570 (55 mg) and TDI-8246 (100 mg) were prepared using the methods as described previously (ref. ^25^). A minor modification was made to the method to obtain intermediates of TDI-6570 in multigram quantities from the reaction mixtures; all intermediates were obtained by filtration using water and cold methanol. Purity of the products was confirmed by performing liquid chromatography–mass spectrometry analysis.

### Production of research diets containing TDI-6570

Research diet pellets containing TDI-6570 (150, 300 or 600 mg of drug per kg diet) were prepared by Research Diet (RDI). As an example, to prepare 150 mg of TDI-6570 per kg diet, all the ingredients were weighed within ±0.5% of the specified weight, and a mixture of TDI-6570 with some sucrose containing a dye (for visualization) was added slowly to the rest of the ingredients. The mixture was homogeneously blended according to the RDI’s proprietary procedures and pelleted. The control diet pellets were prepared similarly without the drug (TDI-6570), and both the TDI-6570 and control diet pellets were dried at 29.5 °C for 2 d and irradiated at 38–43 °C for 40–45 min. The diet pellets were used before the expiration dates tentatively set for 6 months after the date of diet preparation.

### Pharmacokinetic evaluation of TDI-6570

To test pharmacokinetics, TDI-6570 (50 mg per kg (body weight) as a suspension in 0.5% methylcellulose solution in water containing 0.2% Tween 80) was administered to 8-week-old CD-1 male mice (*n* = 3 for each time point) intraperitoneally, and both plasma (EDTA-K2) and brain tissues were collected at various times (0.5, 2, 4, 8 and 24 h after drug administration). Transcardial perfusions were performed with saline before brain collection. Bioanalysis of brain tissue and plasma extracts was performed by liquid chromatography–tandem mass spectrometry.

### Randomization

In the Morris water maze test, all mice from four genotypes were randomly assigned a new number. The hidden platform training and probe trails were conducted by new numerical order. The samples in all in vivo experiments (electrophysiology, snRNA-seq and immunohistochemistry) were randomly allocated into experimental groups in an age-matched and sex-matched manner. For in vitro experiments, control and treatment groups were plated randomly in wells and plates to account for the location effects.

### Blinding

Experimenters were blinded of genotypes when performing image analysis and quantification for all immunohistochemistry quantification experiments. Experimenters were blinded to group allocation during data collection and/or analysis for all of the in vivo experiments (Morris water maze test, electrophysiology and snRNA-seq). For snRNA-seq experiments, the samples were assigned IDs and processed blindly. We decoded the sample identities when we integrated the datasets. For in vitro experiments, codes were assigned to different treatments, samples were collected and analyzed, and the samples were decoded at the end of experiment.

### Statistical analysis

Statistical analyses were performed using R v4.1.0 or GraphPad Prism 8 and 9 as indicated in the legends and Methods. No statistical methods were used to predetermine sample sizes, but our sample sizes are similar to those reported in previous publications^27,66^. Data distribution was assumed to be normal, but this was not formally tested.

### Reporting summary

Further information on research design is available in the Nature Portfolio Reporting Summary linked to this article.

## Online content

Any methods, additional references, Nature Portfolio reporting summaries, source data, extended data, supplementary information, acknowledgements, peer review information; details of author contributions and competing interests; and statements of data and code availability are available at 10.1038/s41593-023-01315-6.

## Supplementary information

Supplementary Information Supplementary Figs. 1–3.

Reporting Summary

Supplementary Table 1 DEGs in *P301S* versus non-transgenic hippocampi. Benjamini–Hochberg-adjusted *P* values reported were calculated using DeSeq2 (v.1.26.0). IPA upstream regulator prediction for DEGs upregulated in *P301S* transgenic hippocampi. Overlapping *P* value was calculated using Fisher’s exact test.

Supplementary Table 2 Gene expression modules derived from weighted gene correlation analyses (WGCNA) on temporal *P301S* bulk-seq data.

Supplementary Table 3 Clinical information for western blotting (refer to Fig. 1i,j).

Supplementary Table 4 DEGs in tau-treated versus control/TDI-8246 + tau versus DMSO + tau treatment iPSC-derived microglia. DEGs induced by tau that were reversed by TDI-8246 treatment. FDR-adjusted *P* values were calculated using MAST (1.18.0).

Supplementary Table 5 DEGs in microglial clusters of *P301S Cgas*^+/−^ versus *P301S Cgas*^+/+^ mice and *P301S Cgas*^*−/−*^ versus *P301S Cgas*^+/+^ mice. FDR-adjusted *P* values were calculated using MAST (1.18.0). Markers of microglia clusters 1, 2, 3 and 4. Bonferroni-adjusted *P* values were calculated using unpaired, two-tailed Wilcoxon rank-sum tests implemented in Seurat (v.3). Gene lists of disease modules in Monocle trajectory analysis.

Supplementary Table 6 DEGs in astrocyte and oligodendrocyte clusters of *P301S Cgas*^−/−^ versus *P301S Cgas*^+/+^ mice. FDR-adjusted *P* values were calculated using MAST (1.18.0).

Supplementary Table 7 DEGs in EN and IN clusters of *P301S Cgas*^−/−^ versus *P301S Cgas*^+/+^ mice. FDR-adjusted *P* values were calculated using MAST (1.18.0). Differentially expressed MEF2C target genes in EN and IN clusters of *P301S Cgas*^−/−^ versus *P301S Cgas*^+/+^ mice.

Supplementary Table 8 Expression values of *MEF2C* in EN and INs. *RNF213*, *IRF3* and *CGAS* in microglia of individuals with AD.

Supplementary Table 9 DEGs in microglia and EN clusters of DMXAA- versus control-treated WT or *Ifnar1*^−/−^ mice. FDR-adjusted *P* values were calculated using MAST (1.18.0). Differentially expressed MEF2C target genes in EN clusters of WT DMXAA- versus control-treated mice.

Supplementary Table 10 In silico calculated pharmacokinetic parameters for TDI-6570. Mean pharmacokinetic parameters for TDI-6570 determined in vivo.

Supplementary Table 11 DEGs in EN and IN clusters of *P301S* TDI versus *P301S* vehicle mice. FDR-adjusted *P* values were calculated using MAST (1.18.0). Differentially expressed MEF2C target genes in EN and IN clusters of *P301S* TDI versus *P301S* vehicle mice.

## Data Availability

All data associated with this study are in the paper or the Supplementary Information. All RNA-seq data have been deposited to the Gene Expression Omnibus under accession number GSE226385. Source data are provided with this paper.

## Source data

Source Data Fig. 1 Unprocessed western blots related to Fig. 1.
Source Data Fig. 2 Unprocessed western blots related to Fig. 2.

## Code availability

All custom code used for snRNA-seq data analysis have been archived at Zenodo (10.5281/zenodo.7717041) and are directly available at https://github.com/lifan36/Udeochu-Amin-Huang-et-al-2023.
