# Supplementary material for: Tau activation of microglial cGAS–IFN reduces MEF2C-mediated cognitive resilience
Source: Nat Neurosci. Author manuscript; Available in PMC 2023 May 30. (PMC10166855; doi:10.1038/s41593-023-01315-6)
Supplement: Supplementary Figures [file NIHMS1897713-supplement-Supplementary_Figures.pdf]

nature neuroscience

Article https://doi.org/10.1038/s41593-023-01315-6

Tau activation of microglial cGAS–IFN reduces MEF2C-mediated cognitive resilience

In the format provided by the authors and unedited

Supplementary Figure 1. Mouse hippocampi single nuclei isolation and RNA sequencing quality control (Related to Figures 3 and 5)

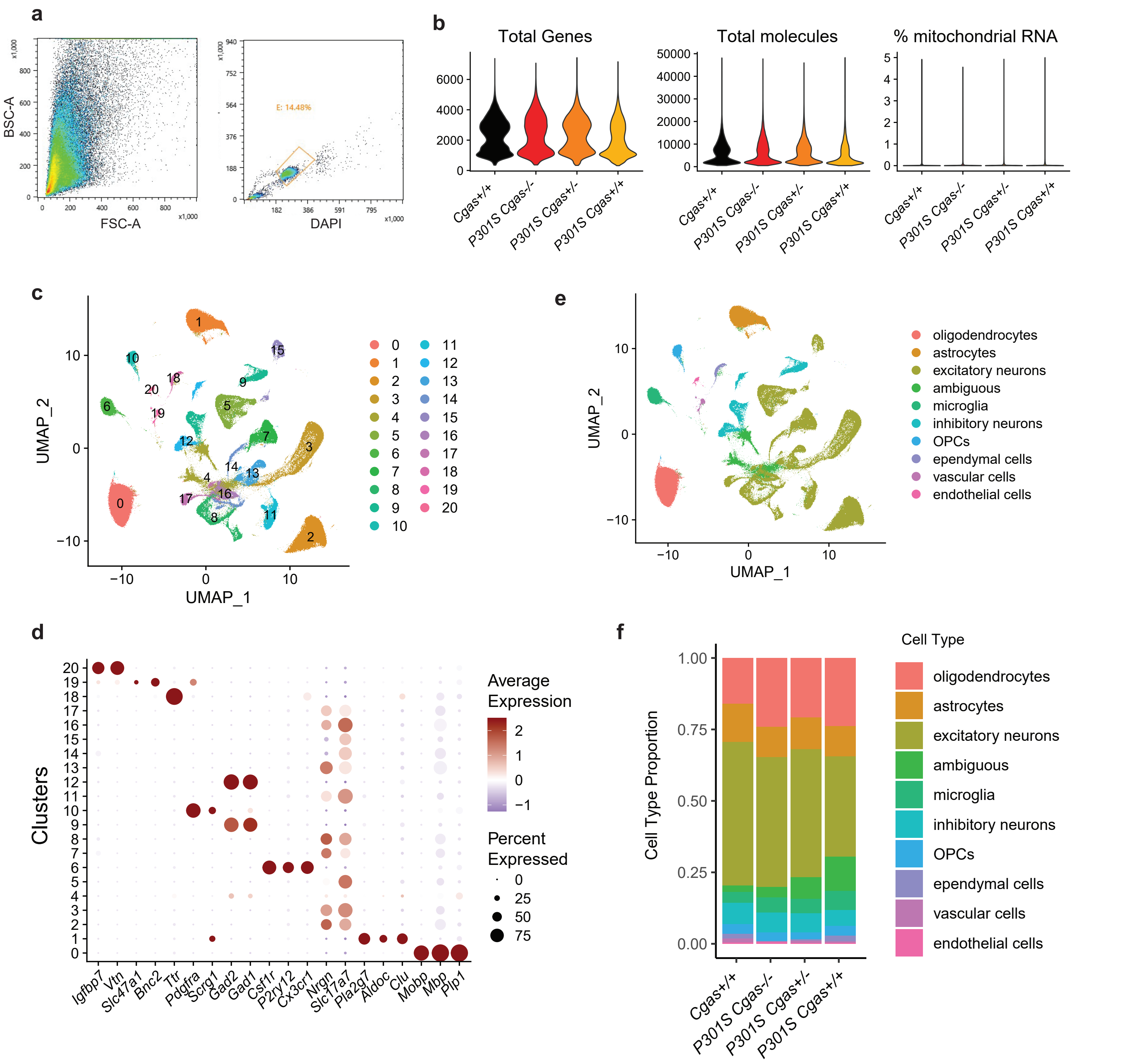

Supplementary Figure 1 | Mouse hippocampi single nuclei isolation and RNA sequencing quality control (Related to Figures 3 and 5). **a.** Representative FACS plot showing gating strategy used for collection of DAPI-labeled hippocampal nuclei (gate E, orange) after mechanical dounce homogenization. n=6 (3 males, 3 females) per genotype. **b.** Quality-control plots showing equivalent amounts of total number of genes, total number of molecules and percent mitochondrial RNA in nuclei used for downstream analyses. **c.** UMAP dimensional plot showing nuclei colored according to transcriptionally distinct cell clusters identified using Seurat package. **d.** Summary of genes used for cluster classification into different cell types. **e.** UMAP plot showing clusters with assigned cell type based in marker expression in (d). **f.** Proportion of each cell type detected across the different genotypes.

Supplementary Figure 2. Quality-control Assessment of snRNA-seq of Control and DMXAA treated WT and *Ifnar1-/-* mice. (Related to Figure 6)

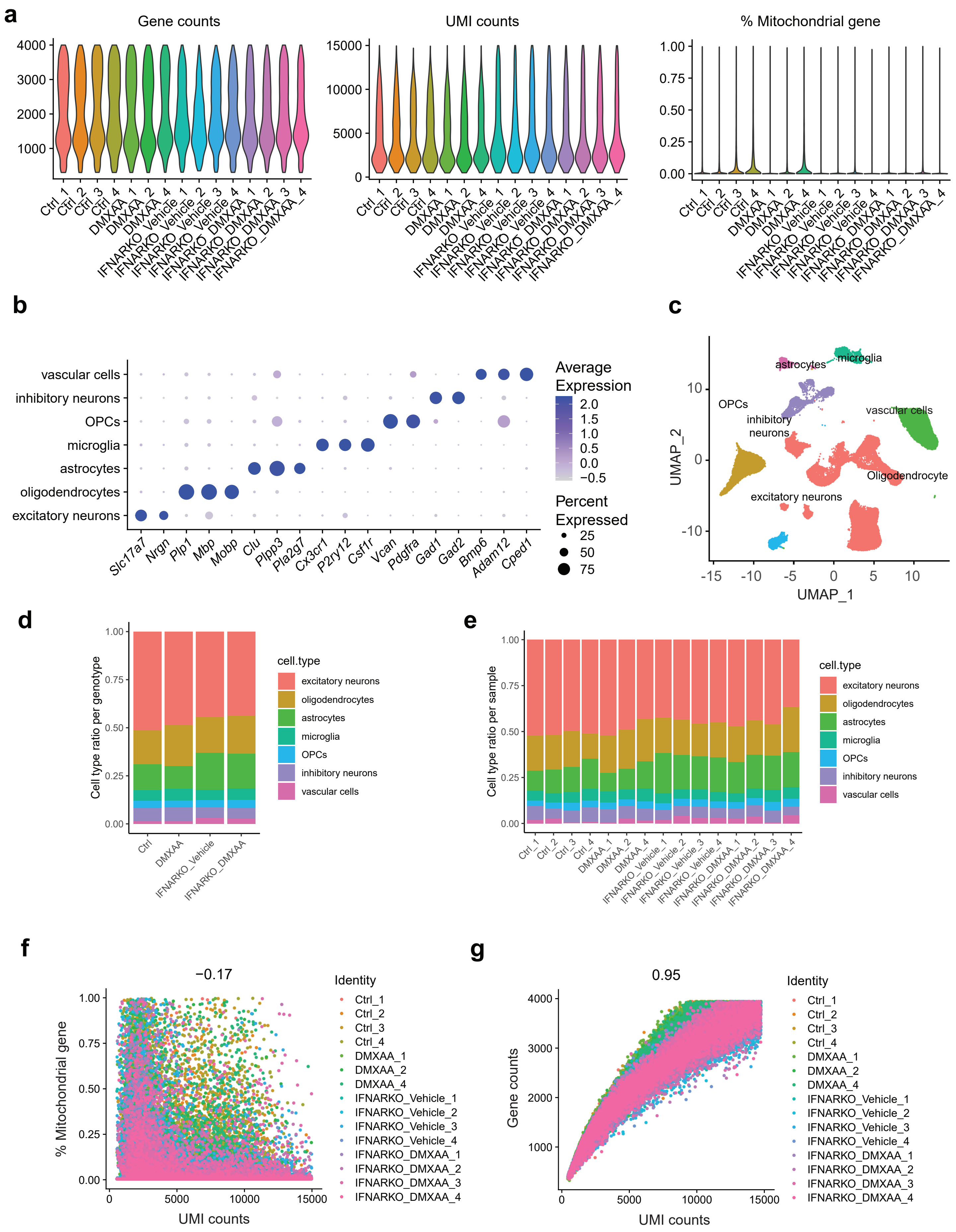

Supplementary Figure 2 | Quality-control Assessment of snRNA-seq of control and DMXAA treated WT and *Ifnar1*$^{-/-}$ mice. (Related to Figure 6). a. Quality-control plots showing equivalent amounts of total number of genes, total number of molecules, and percent mitochondrial RNA in nuclei used for downstream analyses. **b.** Summary of genes used for cluster classification into different cell types. **c.** UMAP dimensional plot showing nuclei colored according to transcriptionally distinct cell clusters identified using Seurat package. **d.** Proportion of each cell type detected across the different genotypes. **e.** Proportion of each cell type detected across the different samples. **f–g.** Correlation between UMI counts and percentage of mitochondrial genes per nuclei (f) and total genes detected (g) for all samples.

Supplementary Figure 3. Quality-control of snRNA-seq of *P301S* hippocampi treated with TDI-6570 (Related to Figure 7)

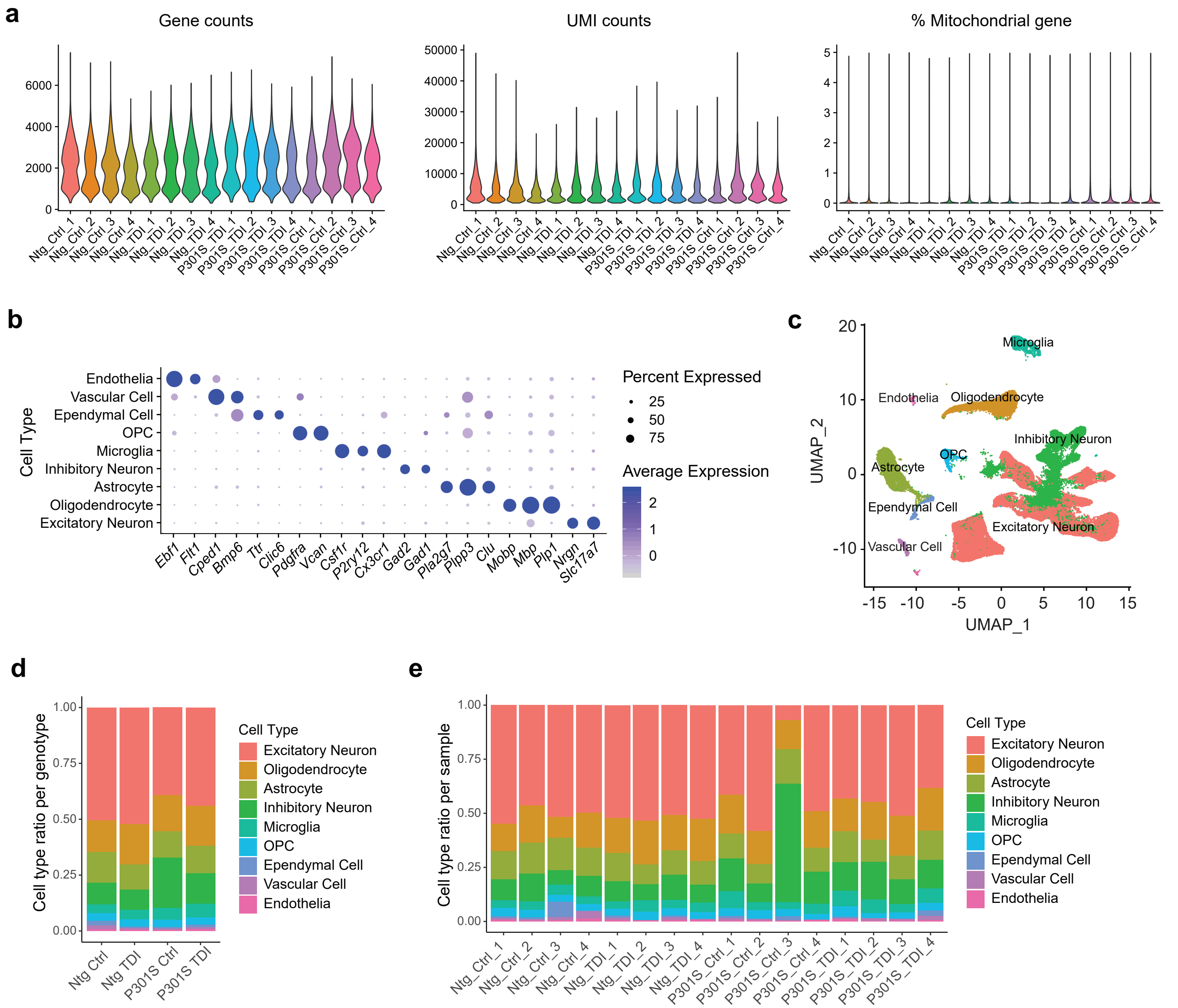

Supplementary Figure 3 | Quality-control of snRNA-seq of *P301S* hippocampi treated with TDI-6570 (Related to Figure 7). a. Quality-control plots showing equivalent amounts of total number of genes, total number of molecules, and percent mitochondrial RNA in nuclei used for downstream analyses. **b.** Summary of genes used for cluster classification into different cell types. **c.** UMAP dimensional plot showing nuclei colored according to transcriptionally distinct cell clusters identified using Seurat package. **d–e.** Proportion of each cell type detected across the different genotypes (d) and each mouse (e). P301S_Ctrl_3 is an outlier thus removed from downstream analyses.
