# Supplementary material for: Tau activation of microglial cGAS–IFN reduces MEF2C-mediated cognitive resilience
Source: Nat Neurosci. 2023 Apr 24;26(5):737–50. doi: 10.1038/s41593-023-01315-6 (PMC10166855; doi:10.1038/s41593-023-01315-6)
Supplement: Supplementary file 1 [file 41593_2023_1315_MOESM14_ESM.pdf]

Source data figure 1. Uncropped western blot scans in Figure 1.

Uncropped western blot scans in Figure 1e.

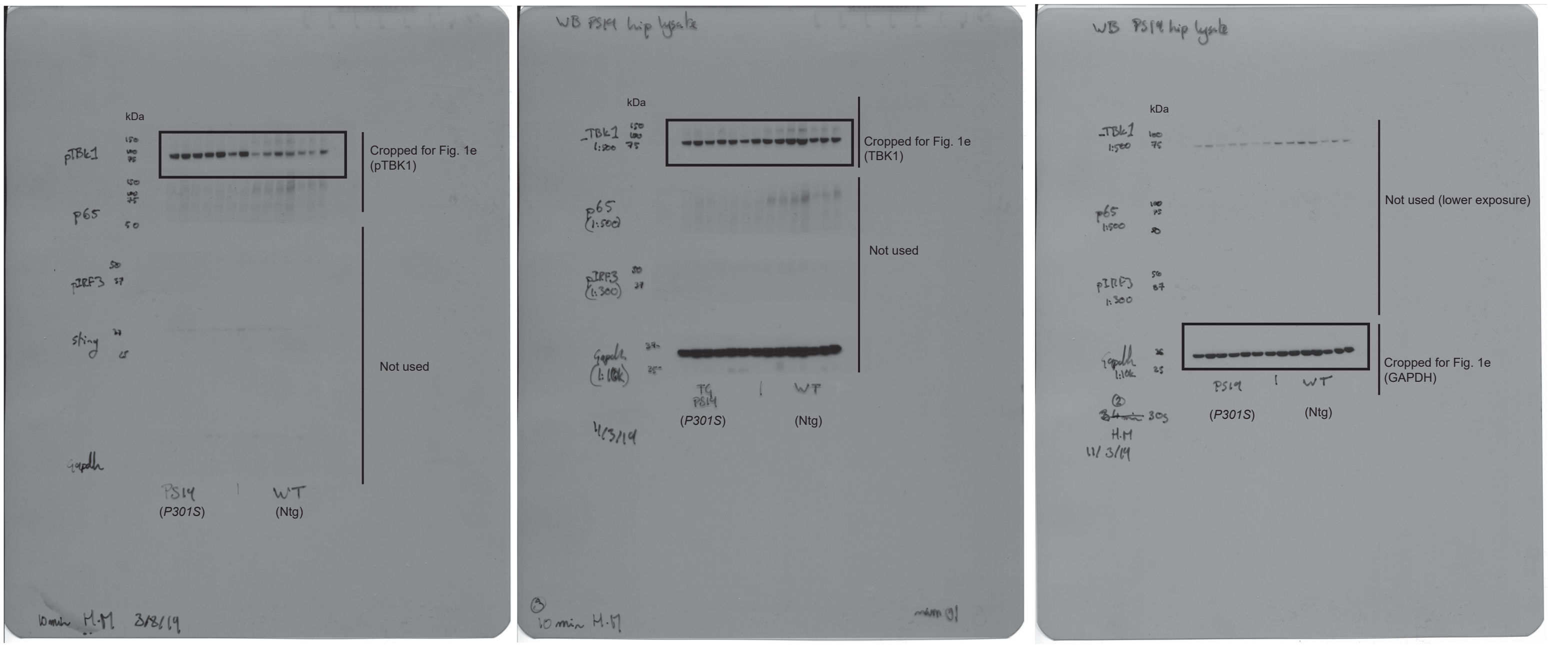

Uncropped western blot scans in Figure 1i.

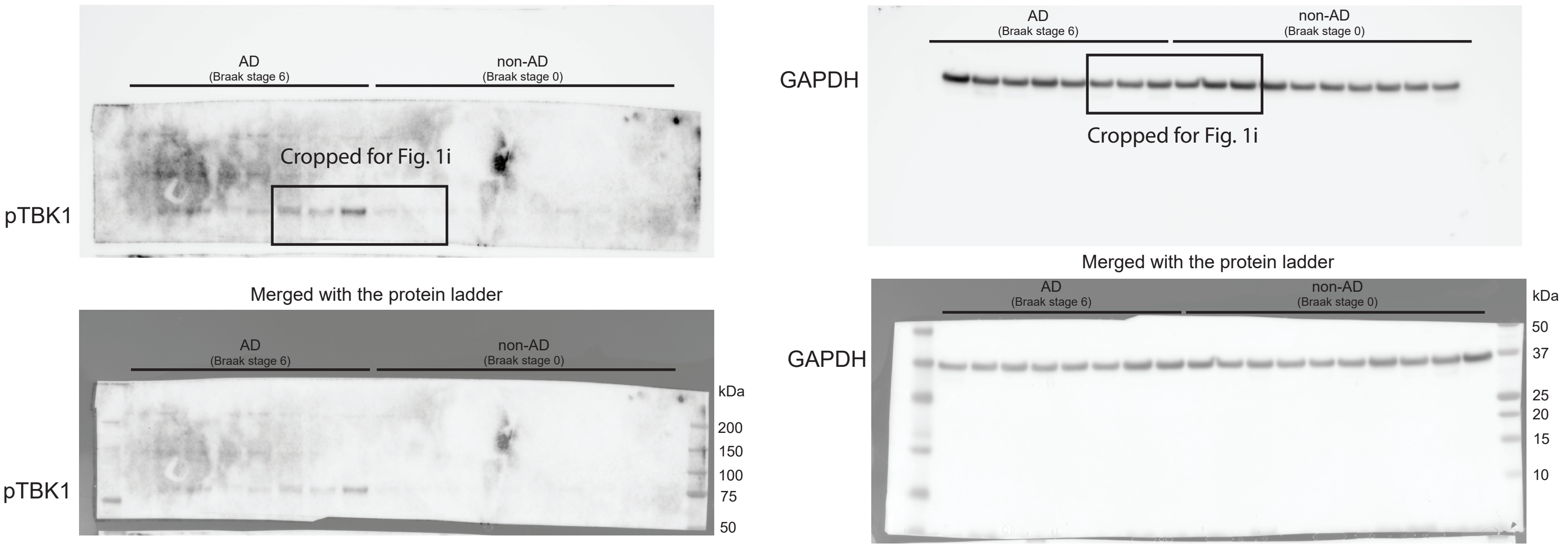

All lanes used in quantification in Fig. 1j
