# Supplementary material for: Tau activation of microglial cGAS–IFN reduces MEF2C-mediated cognitive resilience
Source: Nat Neurosci. 2023 Apr 24;26(5):737–50. doi: 10.1038/s41593-023-01315-6 (PMC10166855; doi:10.1038/s41593-023-01315-6)

Source data figure 2. Uncropped western blot scans in Figure 2.

Uncropped western blot scans in Figure 2b.

Used in the figure

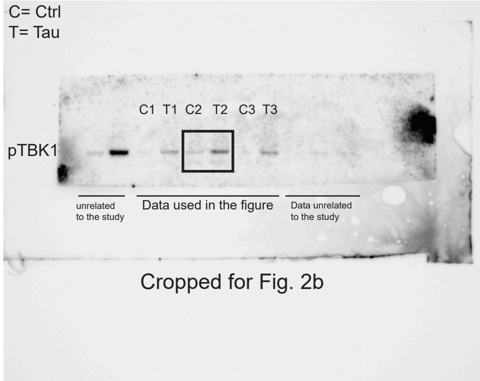

Used in the figure

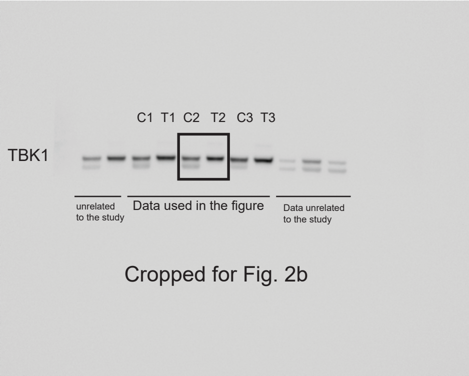

Used in the figure

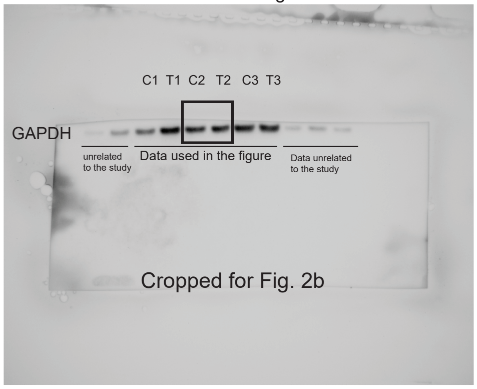

Merged with the protein ladder

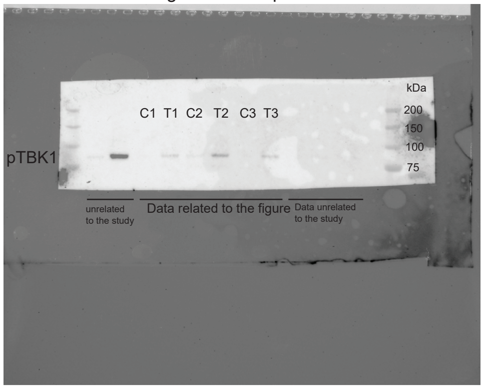

Merged with the protein ladder

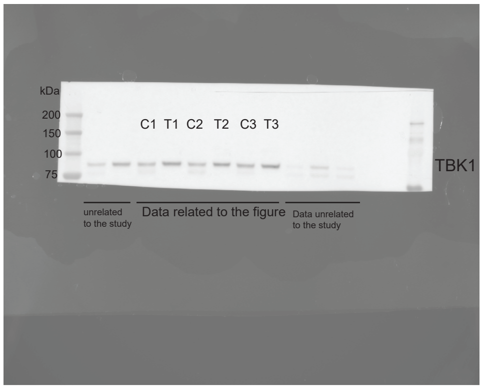

Merged with the protein ladder

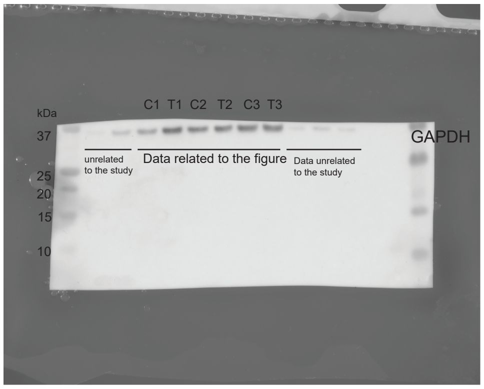

Uncropped western blot scans in Figure 2i.

Used in the figure

Merged with the protein ladder

TOMM20

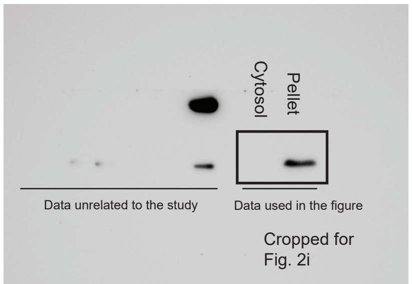

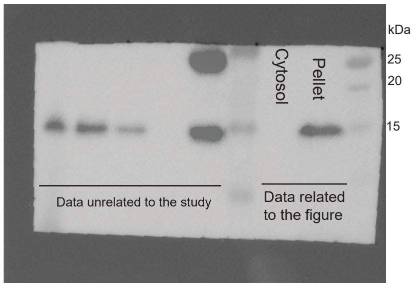

GAPDH

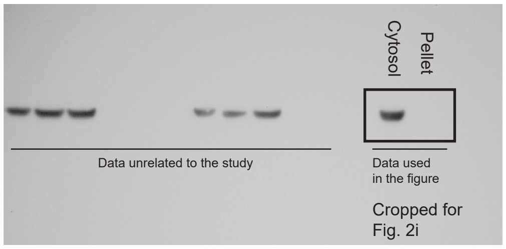

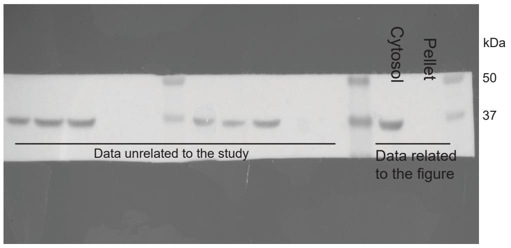

LAMIN A/C

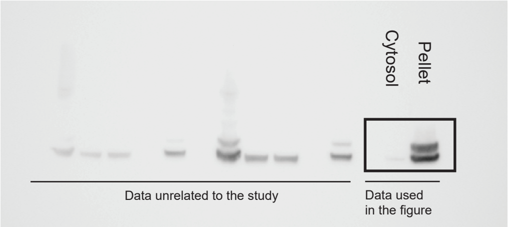

Cropped for Fig. 2i

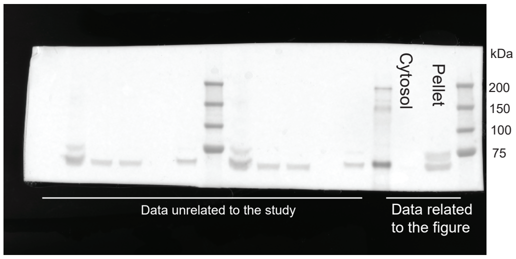

Supplement: Supplementary file 2 [file 41593_2023_1315_MOESM15_ESM.pdf]
