# Supplementary material for: Tau activation of microglial cGAS–IFN reduces MEF2C-mediated cognitive resilience
Source: Nat Neurosci. 2023 Apr 24;26(5):737–50. doi: 10.1038/s41593-023-01315-6 (PMC10166855; doi:10.1038/s41593-023-01315-6)
Supplement: Supplementary file 3 [file 41593_2023_1315_MOESM2_ESM.pdf]

nature portfolio

Corresponding author(s): Li Gan
Sadaf Amin

Last updated by author(s): Mar 10, 2023

Reporting Summary

Nature Portfolio wishes to improve the reproducibility of the work that we publish. This form provides structure for consistency and transparency in reporting. For further information on Nature Portfolio policies, see our Editorial Policies and the Editorial Policy Checklist.

Statistics

For all statistical analyses, confirm that the following items are present in the figure legend, table legend, main text, or Methods section.

n/a	Confirmed	
☐	☒	The exact sample size (*n*) for each experimental group/condition, given as a discrete number and unit of measurement
☐	☒	A statement on whether measurements were taken from distinct samples or whether the same sample was measured repeatedly
☐	☒	The statistical test(s) used AND whether they are one- or two-sided<br>*Only common tests should be described solely by name; describe more complex techniques in the Methods section.*
☒	☐	A description of all covariates tested
☐	☒	A description of any assumptions or corrections, such as tests of normality and adjustment for multiple comparisons
☐	☒	A full description of the statistical parameters including central tendency (e.g. means) or other basic estimates (e.g. regression coefficient) AND variation (e.g. standard deviation) or associated estimates of uncertainty (e.g. confidence intervals)
☐	☒	For null hypothesis testing, the test statistic (e.g. *F*, *t*, *r*) with confidence intervals, effect sizes, degrees of freedom and *P* value noted<br>*Give P values as exact values whenever suitable.*
☒	☐	For Bayesian analysis, information on the choice of priors and Markov chain Monte Carlo settings
☒	☐	For hierarchical and complex designs, identification of the appropriate level for tests and full reporting of outcomes
☒	☐	Estimates of effect sizes (e.g. Cohen's *d*, Pearson's *r*), indicating how they were calculated

Our web collection on statistics for biologists contains articles on many of the points above.

Software and code

Policy information about availability of computer code

Data collection	No software was used to collect data.
Data analysis	All custom codes used for snRNA-seq data analysis have been archived at Zenodo (https://doi.org/10.5281/zenodo.7717041) and are directly available at https://github.com/lifan36/Udeochu-Amin-Huang-et-al-2023.<br>ImageJ Software (v.2.1.0) (NIH) was used for image processing and analysis. Bulk RNA-seq reads were mapped using the BlueBee genomics platform and STAR program. The read count table was generated with the RSEM program. For single nuclei RNAseq, gene counts were obtained by aligning reads to the mouse genome (mm10) with Cell Ranger software 3.1.0. Potential doublet cells were predicted using DoubletFinder. Normalization and clustering were done with the Seurat package v_3.2.2). DEGS were analysed using (MSigDB) gene annotation database (https://www.gsea-msigdb.org/gsea/msigdb/) and QIAGEN's Ingenuity Pathway Analysis (IPA, QIAGEN Redwood Coty, www.qiagen.com/ingenuity). Statistical Analyses were performed with Graphpad Prism (v_8 & v_9.0, Graphpad, San Diego, California) and R (v_4.1.0, R Foundation for Statistical Computing, Vienna, Austria). Data Visualization was done with Graphpad and R package ggplot 2 (v_3.3.6).

For manuscripts utilizing custom algorithms or software that are central to the research but not yet described in published literature, software must be made available to editors and reviewers. We strongly encourage code deposition in a community repository (e.g. GitHub). See the Nature Portfolio guidelines for submitting code & software for further information.

Data

Policy information about availability of data

All manuscripts must include a data availability statement. This statement should provide the following information, where applicable:

- Accession codes, unique identifiers, or web links for publicly available datasets
- A description of any restrictions on data availability
- For clinical datasets or third party data, please ensure that the statement adheres to our policy

All data associated with this study are in the paper or the Supplementary materials. All RNA-seq data have been deposited to the Gene Expression Omnibus (GEO) under accession number GSE226385. Unedited and uncropped scans of all blots are supplied in the source data files.

Human research participants

Policy information about studies involving human research participants and Sex and Gender in Research.

Reporting on sex and gender	De-identified human postmortem brain samples were obtained from UCSD and Mount Sinai NIH Brain and Tissue Repository. Samples from male (n=6) and female (n=12) subjects were used in the study. The gender information for each sample is reported in the supplementary table 3.
Population characteristics	The information for age, Braak stage and clinical diagnosis and postmortem interval (PMI) is provided for all human brain samples in supplementary table 3.
Recruitment	The participants were recruited in accordance with the above-mentioned Biobank recruitment strategies. For the current study, the brain samples were chosen based on Braak stage (0, 1 or 6).
Ethics oversight	The collection and banking of the samples were carried out in accordance with the Institutional Review Board (IRB) protocols for the respective Brain banks.

Note that full information on the approval of the study protocol must also be provided in the manuscript.

Field-specific reporting

Please select the one below that is the best fit for your research. If you are not sure, read the appropriate sections before making your selection.

☒ Life sciences ☐ Behavioural & social sciences ☐ Ecological, evolutionary & environmental sciences

For a reference copy of the document with all sections, see nature.com/documents/nr-reporting-summary-flat.pdf

Life sciences study design

All studies must disclose on these points even when the disclosure is negative.

Sample size	The sample size for in vivo and in vitro experiments was determined on the basis of previous publications and our experience. For the studies in live animals, the number of animals was determined by power analysis using information from similar studies published by our group or others. For studies involving brain section analyses, the number of slices was determined based on our previous studies to account for variabilities. For in vitro experiments, we determined the experimental replications to account for the technical variabilities and changes with treatment based on our experience. No statistical methods were used to predetermine sample sizes but our sample sizes are similar to those reported in previous publications (PMID: 35413950, PMID: 34851693).
Data exclusions	In our analyses of snRNA-seq data, we excluded the data from potential doublet cells as identified by DoubletFinder. For Cgas genetic cohort snRNA-seq, we sequenced and integrated samples from Cgas+/+ (n=5), Cgas+/- (n=2), Cgas-/- (n=2), P301S Cgas+/+ (n=6), P301S Cgas+/– (n=6), and P301S Cgas–/– (n=6) mice. Cgas+/- and Cgas-/- genotypes had sample size of 2 and thus were not included in downstream analyses. In analysis of snRNA-seq for TDI vs control diet fed mice, one mouse (P301S_Ctrl_3) was identified as an outlier and excluded.
Replication	All in vitro experiments were performed with a minimum of 3 biological replicates. All in vivo experiments involving behavioral tests and pathology analysis were performed with a minimum of 4 mice per genotype. The samples for snRNAseq were 2-4 mice per genotype. All attempts at replication were successful when a representative data set is provided.
Randomization	In Morris water maze test, all mice from four genotypes were randomly assigned a new number. The hidden platform training and probe trails were conducted by new numerical order. The samples in all in vivo experiments (Electrophysiology, snRNAseq, IHC) were randomly allocated into experimental groups in an age-matching and sex-matching manner. For in vitro experiments, control and treatment groups were attributed randomly on wells and plates to account for the location effects.
Blinding	Experimenters were blinded of genotypes when performing image analysis and quantification for all IHC quantification experiments. Experimenters were blinded to group allocation during data collection and/or analysis for all of our in vivo experiments (Morris water maze test, electrophysiology, snRNAseq). For in vitro experiments, codes were assigned to different treatments, samples were collected, and

analyzed and the samples were decoded at the end of experiment. For RNAseq experiments, the samples were assigned IDs and then processed blindly. We decoded the sample identities when we integrated the datasets.

Reporting for specific materials, systems and methods

We require information from authors about some types of materials, experimental systems and methods used in many studies. Here, indicate whether each material, system or method listed is relevant to your study. If you are not sure if a list item applies to your research, read the appropriate section before selecting a response.

Materials & experimental systems

n/a	Involved in the study
☐	☒ Antibodies
☐	☒ Eukaryotic cell lines
☒	☐ Palaeontology and archaeology
☐	☒ Animals and other organisms
☒	☐ Clinical data
☒	☐ Dual use research of concern

Methods

n/a	Involved in the study
☒	☐ ChIP-seq
☐	☒ Flow cytometry
☒	☐ MRI-based neuroimaging

Antibodies

Antibodies used

Antibodies used in immunofluorescence analysis were as follows: Secondary antibodies used were Alexa fluor donkey anti-rabbit/goat 488 and anti-mouse 555, and Jackson donkey anti-goat 555 at 1:500 (Invitrogen).
STING (Clone D2P2F, Cell Signaling Technology, 13647, 1:300), anti-IBA1 (Abcam, ab5076, 1:500), anti-PSD-95 (Clone 6G6-1C9, Millipore, MAB1596, 1:500), antivGAT (Millipore, Ab5062, 1:500), anti-pSTAT1 (Clone 58D6, Cell Signaling, mAb9167, 1:500), anti-NRG1 (Clone 7D5, Invitrogen, MA5-12896, 1:100), MC1 (a generous gift from Dr. Peter Davis), anti-MEF2C (Clone 681824, R&D systems, MAB6786, 1:200) , anti-NeuN (Millipore, ABN78, 1:500), anti-gamma-H2.AX (abcam, ab2893, 1:500).
Antibodies used in western blot were as follows: TBK1 (Clone D1B4, Cell Signaling Technology, 3504, 1:1000), pTBK1 (Clone D52C2, Cell Signaling Technology, 5483, 1:500), GAPDH (Clone 6C5, Millipore, MAB374, 1:10000 and GeneTex, GTX100118, 1:10000), TOMM20 (Clone 4F3, Millipore Sigma, ST1705, 1:1000), Lamin A/B1/C (Clone EPR4068, abcam, ab108922, 1:1000).
Secondaries used were anti-rabbit HRP (Calbiochem, 401393, 1:2000) or anti-mouse HRP (Calbiochem, 401253, 1:2000)
For Immuno-gold labeling electron microscopy antibody against tau (Agilent Technologies, A0024, 1:1000) was used.

Validation

Each antibody was validated for the species (mouse or human) and application (immunohistochemistry, flow cytometry, immunoprecipitation) by the correspondent manufacturer. The validation studies can be found on the manufacturer's website. The usage was described in full detail the methods section of the manuscript. We further confirmed the STING antibody (D2P2F, Cell Signaling Technology) for use in immunohistochemistry by staining wild type and STING KO (Jackson Laboratory, Strain #: 017537). The confirmation data is not included in the manuscript.

Eukaryotic cell lines

Policy information about cell lines and Sex and Gender in Research

Cell line source(s)

BV2 cells were purchased from Banca Biologica e Cell Factory - IRCCS AOU San Martino - IST Genova.
THP1-Dual™ Cells (Cat. number: thpd-nfis) and THP1-Dual™ KO-cGAS Cells (Cat. number: thpd-kocgas) were purchased from InvivoGen.

Authentication

Cell line authentication was performed by the supplier, but not independently authenticated in our lab. The supplier performed validation assays with Multiplex PCR: tested against human, rat, mouse primers. Confirmed as mouse with cytochrome c oxidase subunit I (cox I) primers.

Mycoplasma contamination

Cell lines were routinely tested for Mycoplasma contamination using a mycoplasma detection kit. No mycoplasma contamination was detected in the samples tested.

Commonly misidentified lines
(See ICLAC register)

This manuscript does not involve experiments with any of the commonly misidentified lines.

Animals and other research organisms

Policy information about studies involving animals; ARRIVE guidelines recommended for reporting animal research, and Sex and Gender in Research

Laboratory animals

Mice were housed no more than 5 per cage, given ad libitum access to food and water, and were housed in a pathogen-free barrier facility with 70-74°F, 30-70% humidity a 12 h light-dark cycle. P301S transgenic mice (https://www.jax.org/strain/008169) were crossed with Cgas-/- mice (https://www.jax.org/strain/026554) to generate P301S Cgas+/- mice, and subsequent crossing of F1 litters generated Cgas+/+, Cgas+/- and Cgas-/- mice and their corresponding P301S transgenic littermates. Mice of both sexes were used for

behavioral, histological and biochemical analyses. Mice underwent behavioral testing at 7 to 8 months of age and had not been used for any other experiments. At 9 to 10 months of age, the same mice were used for pathology and RNA-seq studies. For TDI-6570 in vivo treatment, P301S and non-transgenic littermate mice at 6-7 months were used for diet experiments and were assayed for behavior and histology at 9-10 months. Wild type C57BL/6J (https://www.jax.org/strain/000664) and Ifnar1-/- (https://www.jax.org/strain/028288) mice were purchased from the Jackson Laboratory.

Wild animals	No wild animals were used in the study.
Reporting on sex	Mice of both sexes were used for behavioral, histological and biochemical analyses.
Field-collected samples	No field-collected samples were used in the study.
Ethics oversight	All animal work was performed in accordance with NIH guidelines and all mouse protocols were approved by the Institutional Animal Care and Use Committee, University of California, San Francisco and Weill Cornell Medicine.

Note that full information on the approval of the study protocol must also be provided in the manuscript.

Flow Cytometry

Plots

Confirm that:

☒ The axis labels state the marker and fluorochrome used (e.g. CD4-FITC).

☒ The axis scales are clearly visible. Include numbers along axes only for bottom left plot of group (a 'group' is an analysis of identical markers).

☒ All plots are contour plots with outliers or pseudocolor plots.

☒ A numerical value for number of cells or percentage (with statistics) is provided.

Methodology

Sample preparation	In brief, mouse brain tissue was placed in 1500 µl of Sigma nuclei PURE lysis buffer (Sigma, NUC201-1KT) and homogenized with a Dounce tissue grinder (Sigma, D8938-1SET) with 15 strokes with pestle A and 15 strokes with pestle B. The homogenized tissue was filtered through a 35-µm cell strainer and were centrifuged at 600 g for 5 min at 4°C and washed three times with 1 ml of PBS containing 1% BSA, 20 mM DTT and 0.2 U µl−1 recombinant RNase inhibitor. Then the nuclei were centrifuged at 600 g for 5 min at 4°C and re-suspended in 500 µl of PBS containing 0.04% BSA and 1x DAPI.
Instrument	Sony Corporation, LE-MA900FP
Software	Sony Corporation, Cell Sorter Software v3.2
Cell population abundance	14.48% of total events were DAPI+ nuclei using DAPI-A vs DAPI-H.
Gating strategy	Most events were included in the FSC/SSC gate. Singlet, DAPI-stained nuclei were sorted using DAPI-A vs DAPI-H.

☒ Tick this box to confirm that a figure exemplifying the gating strategy is provided in the Supplementary Information.
